# Dopaminergic short axon cells integrate sensory and top–down inputs to enhance discriminative learning in the mouse olfactory bulb

**DOI:** 10.1371/journal.pbio.3003375

**Published:** 2025-09-16

**Authors:** Rahul Garg, Vikas Kumar, Jae Hyoun Seiler, Yunming Wu, Melainia McClain, Kexi Yi, C. Ron Yu

**Affiliations:** 1 Graduate School of Stowers Institute for Medical Research, Kansas City, Missouri, United States of America; 2 Stowers Institute for Medical Research, Kansas City, Missouri, United States of America; 3 Department of Neurosciences, Case Western Reserve University School of Medicine, Cleveland, Ohio, United States of America; 4 Department of Biology, Howard Hughes Medical Institute, Stanford University, Stanford, California, United States of America; 5 Department of Physiology and Cell Biology, University of Kansas Medical Center, Kansas City, Kansas, United States of America; Okinawa Institute of Science and Technology Graduate University: Okinawa Kagaku Gijustu Daigakuin Daigaku, JAPAN

## Abstract

Discriminative learning enhances contrast between sensory inputs to allow fast and accurate decision-making. However, the neural mechanisms that selectively enhance sensory representations to improve discrimination remain unclear. Here, we show that learning-induced differential gating of olfactory inputs takes place at the first stage of sensory processing in the mouse olfactory bulb and requires dopaminergic short axons cells (SACs). Optical imaging, spatial transcriptomics, and electron microscopy experiments reveal that synaptic and structural plasticity in SACs allows odor valence-based modulation of their interactions with other cell types in the olfactory glomeruli. Importantly, an increase in tyrosine hydroxylase expression by SACs surrounding responding glomeruli, with a bias towards those activated by reward odors, creates a valence-based modulation of sensory input. Further, we identify cholinergic input from the horizontal limb of the diagonal band as the valence-dependent signal that modulates SAC activities and refines sensory representation via disinhibition. Our findings reveal a circuit mechanism where an interneuron population serves as a central hub integrating sensory input and top–down signal to enhance sensory acuity.

## Abstract 2

Discriminative learning enhances sensory contrast for rapid, accurate decisions, yet the neural basis for this remains unclear. This study describes a sensory-forebrain circuit centered on dopaminergic short axon cells in the mouse olfactory bulb, which refines sensory input during learning.

## Introduction

Sensory experience can induce both short-term and long-term changes in the brain that enable animals to adapt to their environment. During early postnatal development, exposure to sensory stimuli profoundly influences neuronal connectivity in sensory areas. These experiences shape neural circuits to capture the statistical properties of sensory input, enabling animals to efficiently discriminate between stimuli in their environment [1–10]. In adults, however, large-scale structural plasticity is limited, and adaptive changes primarily occur through synaptic mechanisms such as spike timing dependent plasticity, which can influence behavior over days [11–18]. Both exposure-driven and reinforcement-based learning can engage these synaptic mechanisms to reshape sensory representations, either through repeated experience or through associations with rewarding or aversive outcomes. It is not clear how these learning processes induce separation of neural patterns to enhance sensory discrimination.

A likely mechanism may rely on neuromodulatory influences on synaptic interactions to enable sensory gating and facilitate learning. Neuromodulators, including acetylcholine, can modulate synaptic efficacy by altering receptor sensitivity, intracellular signaling cascades, or pre- and post-synaptic excitability. Their actions can be exerted directly at synapses in feedforward pathways or indirectly by targeting interneurons, thereby shaping circuit-level processing [19–22]. Crucially, neuromodulation can act on different timescales. It may rapidly modulate synaptic strength in response to immediate sensory stimulation or behavioral demands, through precisely timed activities, or through long-term modification of circuit connectivity [23]. In the olfactory bulb (OB), such modulation is mediated by both bottom–up and top–down inputs. Olfactory sensory neurons (OSNs) expressing the same receptor converge onto the same glomerulus, creating a reproducible spatial map of odor identity [24]. While prior studies have shown that cholinergic input from the basal forebrain can enhance odor sensitivity and influence habituation and reward-related processing [25–27], the circuit-level mechanisms through which acetylcholine shapes early sensory representations to impact behavior remain poorly defined. An earlier study showed that basal forebrain cholinergic activity suppressed spontaneous activities of the periglomerular cell, granule cells, and the mitral/tufted cells, but it remained unclear if the process enhances odor discrimination [27]. Here, we investigate how experience-dependent modulation in the OB enhances odor discrimination.

Odors elicit distinctive patterns of glomerular activity, enabling us to examine changes associated with various forms of learning. We show that discriminative learning leads to differential enhancement of odor-evoked responses, which is directly associated with enhanced behavioral discrimination and decision-making. By subjecting mice to acute exposure, associative learning, and discriminative learning paradigms, we identify molecular and structural changes driven by various experiences. The expression of tyrosine hydroxylase (TH), the key enzyme for dopamine synthesis expressed by short axon cells (SACs) in the olfactory bulb, has been shown to be activity dependent [28,29]. We show that TH expression is differentially regulated by sensory experiences. We also determined that cholinergic signals acted through the SACs to enhance discrimination between different stimuli and improve behavioral outcomes. As the SACs are strategically positioned in the glomerular layer to influence the activity of both OSN terminals and the mitral/tufted cells, the primary output neurons, our findings position the SACs as a crucial unit integrating top–down and bottom–up signals to modulate odor information processing. Our study illustrates the importance of differential modulation of sensory processing in discriminative learning and a mechanism of this control.

## Results

### Discriminatory learning preferentially enhances reward-associated inputs

**Fig 1:**
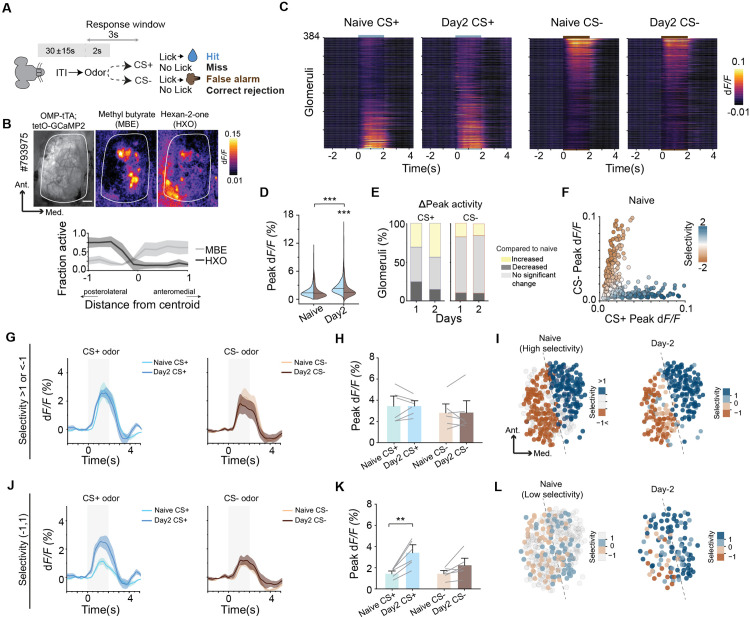
Discriminatory learning preferentially enhances reward associated input. (A) Illustration of odor discrimination training paradigm. (B) Representative dorsal OB plane imaged in an OMP-tTA; tetO-GCaMP2 animal. Scale bar 200 µm. Heatmaps show response to hexan-2-one (HXO) and methyl butyrate (MBE). Bottom: Fraction of responsive glomeruli across the dorsal OB of 6 mice. (C) Heatmaps show dynamic calcium responses of 384 glomeruli from 6 mice across training sessions. Left, responses for CS+ odor (HXO); Right: CS− odor (MBE). Odors were delivered from *t* = 0 to 2 s. (D) Violin plots show response amplitude of all glomeruli imaged before (Naive) and after (day 2) training to CS+ (blue) and CS− (brown) odors. Naive, N.S. (not significant); Day 2, ***P < 0.001, two-way repeated measures ANOVA followed by Tukey’s *post hoc* test. (E) Fraction of glomeruli with significant changes post learning. Yellow: increased, dark grey: decreased, light grey: unchanged. (F) Scatter plot of peak response of individual glomeruli to both odors in naive animals. Colormap represents stimulus selectivity for each glomerulus. (G) Left: Average CS+ odor response of glomeruli highly selective for each odor before (naive; light blue) and after (Day 2; dark blue) training. Right panels: Average CS− odor responses of glomeruli highly selective for odors before (naive; light brown) and after (Day 2; dark brown) training. Shaded areas indicate odor delivery. Data are mean ± standard error. (H) Bar plots showing peak responses for odors in highly selective glomeruli before and after training. N.S, two-way repeated measures ANOVA followed by Tukey’s *post hoc* test. (I) Changes in the responses of high-selectivity glomeruli post training. (J**–**L) Same as **(G–I)** but for low selectivity glomeruli. **P < 0.01, two-way repeated measures ANOVA followed by Tukey’s *post hoc* test. Data for this figure are provided in S1 Data.

In the olfactory discrimination task, mice were trained to differentiate between two odors to earn a water reward and avoid punishment (Fig 1A). Over repeated trials, mice reduced lick initiation events for the CS− odor, resulting in increased task accuracy (S1A and S1B Fig; correct rejection rate for CS− odor increased from 39% to 71% on average from Day 1 to Day 2). We utilized widefield calcium imaging to monitor odor-elicited activity of OSN axon termini from *OMP-tTA; tetO-GCaMP2* mice [30,31]. We used hexan-2-one as the CS+ and methyl butyrate as the CS− odor, or vice versa, and recorded the activity of 384 glomeruli on the dorsal surface of the olfactory bulb in six mice before and after learning (Fig 1B). While imaging access was limited to the dorsal surface, c-Fos staining in odor-exposed mice confirmed significantly greater activation in the dorsal OB compared to medial, lateral, and ventral regions (S1C Fig). The two odors evoked responses from largely spatially distinct sets of glomeruli (MBE: anteromedial and HXO: posterolateral) with overlap on the dorsal bulb [31], enabling comprehensive analysis of response changes across these populations (Figs 1C and S1D). Training preferentially enhanced the response to the CS+ odor over CS− odor (Fig 1D; *q*(383) = 6.58; *p* < 0.0001). This effect can be attributed to an increase in response amplitude but not the percentage of glomeruli responding to CS+ odors (Figs 1E and S1E). As many glomeruli show overlapping but biased response to the odors, we calculated odor selectivity from the differential peak amplitudes to the two odors to examine specific changes of glomerular response during learning (Fig 1F; “Materials and methods”). For glomeruli with high selectivity (>1 or <−1), there were no changes in their response profiles (Fig 1G–1I; *q*(5) = 1.54; *p* = 0.32). For glomeruli with low selectivity ([−1, 1]) to either CS+ or CS− odors, the response to CS+ but not CS− odors were increased (Fig 1J–1L; *q*(5) = 8.28; *p* = 0.002), which can be visualized in the glomerular selectivity maps. Overall, training primarily enhanced glomerular selectivity to CS+ odors (S1F Fig).

Training also accelerated temporal responses. Response onsets were advanced for both CS+ (19% glomeruli with faster onset) and CS− odors (11%; S1G and S1H Fig). Overall, response latency shortening was more significant for the CS+ odors. Faster onsets for both odors were notable in CS+ selective glomeruli (S1I Fig). Taken together, discriminative training selectively enhanced axonal activity in the OB for the CS+ but not CS− odors. As we show later, this effect is the result of modulation by local circuitry in the OB on OSN axon termini. It is not due to a selective increase in response of the OSNs in the olfactory epithelium.

### Discriminative learning alters molecular composition in the olfactory glomeruli

**Fig 2:**
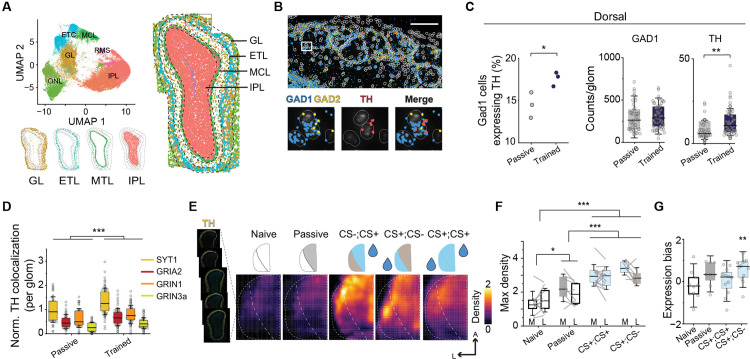
Discriminative learning differentially enhances TH expression by SACs. (A) Cell type identification in the OB by spatial transcriptomics. Top left, cell clusters identified based on gene expression from OB sections shown in UMAP. Bottom left and right panels: location of cell types in the OB as indicated. GL: glomerular layer, MCL: mitral cell layer, IPL: internal plexiform layer. (B) Top: magnified area from the rectangle in **(A)** showing transcripts of GAD1, GAD2, and TH in the dorsal GL. Dashed circles indicate individual glomeruli. Scale bar: 200 µm. Bottom: magnified region from the top panel showing transcripts in individual cells (solid circles). (C) Percentage of TH-expressing GAD1 cell. Middle and right panels: GAD1 and TH transcripts per glomerulus (right) of passively exposed (grey) and trained (dark blue) animals. *P < 0.05, **P < 0.01, two sample *t* test (two-tailed). (D) Bar plots show normalized colocalization of synaptic and TH transcripts per glomerulus in dorsal region. ***P < 0.001, two-way ANOVA followed by Tukey’s *post hoc* test. (E) Spatial density of TH immunofluorescence over the dorsal OB across different training paradigms. (F) Box plots show maximal density observed in odor associated regions (M: medial; L: lateral). *P < 0.05, ***P < 0.001, mixed-design ANOVA followed by Tukey’s *post hoc* test. (G) Spatial bias in TH expression. Values above 0 indicate that expression is biased towards odor stimulation or towards the rewarded odor for discrimination tasks. **P < 0.01, one sample *t* test (two-tailed). Data for this figure are provided in S1 Data.

Odor exposure can lead to transient and long-term adaptation in glomerular activity [32,33] and alter gene expression in the OSNs [34–37]. Sensory deprivation and olfactory training differentially affect gene expression in the OB [38–44]. We hypothesized that learning induces molecular changes in the interneurons in the glomerular layer (GL) to modulate odor responses. To determine the cell and neurotransmitter types that may participate in learning associated modulation, we used Molecular Cartography, a spatial transcriptomics approach based on multiplex single-molecule fluorescent imaging to probe the expression of key neuronal markers in the OB (Fig 2A). This approach allowed us to identify distinct cell types, their precise location, and associated genes in the OB to link specific molecular changes to functional outcomes. Consistent with past findings, we identified a subpopulation of GAD1 expressing cells that also expressed TH (S2A and S2B Fig). There was an increase of these cells in mice undergoing discriminative training when compared to those with passive odor exposure (Fig 2B and 2C; independent sample test: *t*(4) = –3.15, *p* = 0.034, pooled glomeruli test: *t*(101) = –3.2, *p* = 0.0018), either due to an increase in TH-expressing cells or due to previously low-expressing cells now reaching the detection threshold. To investigate whether learning-induced changes in TH expression were accompanied by synaptic adaptations that could support altered circuit function, we next examined the expression of glutamate receptor genes across OB layers (S2C–S2E Fig). In the TH+ cells, we found that genes associated with synaptic plasticity, including synaptotagmin 1 (SYT1), AMPA receptor GRIA2, NMDA receptors GRIN1 and GRIN3a, all increased in dorsal OBs of trained animals (Fig 2D; *q*(400) = 7.66; *p* < 0.0001). These increases were specific to the dorsal region and were not observed in other layers, suggesting that learning selectively enhances synaptic gene expression in dopaminergic SACs within the odor-responsive region. This line of evidence indicates that plasticity of the TH-expressing SACs may play a central role in olfactory learning.

### Discriminative learning differentially enhances TH expression by SACs

We hypothesized that the increase in TH results from the pairing of a reward signal with odor stimulation and predicted that TH expression increases in glomeruli representing rewarded but not unrewarded odors. We tested this hypothesis by examining the pattern of TH expression across training conditions that include passive exposure, non-discriminatory pairing (both odors paired with reward), and discriminatory association (CS+ and CS− pairing). We took advantage of the segregated patterns of glomerular activity evoked by two odors to determine the change of expression. We used immunofluorescent staining on serial sections of the bulb to quantify and construct density maps to reveal the distinctive patterns of TH expression induced by the training paradigms (Figs 2E and S2F). We made two observations with discriminatory training. First, TH expression in the region representing the reward odor was higher compared to those with no exposure or passive exposures (Fig 2F; naïve *q*(36) = 11.82, *p* < 0.0001; passive *q*(36) = 7.07, *p* < 0.0001). Second, the areas representing the CS− odor also showed a significant increase in TH expression when compared to passive exposure (*q*(36) = 4.63, *p* = 0.042). The level of increase was on par with non-discriminatory pairing with rewards compared to passive exposure (*q*(36) = 4.8, *p* = 0.032). We measured the expression bias of TH for each training paradigm (“Materials and methods”). The most pronounced differentials in TH expression were observed in the discriminatory training group (Fig 2G; *t*(11) = 3.61, *p* = 0.004), indicating *t*hat the TH expression was linked not merely to the presence of a rewarded stimulus, but specifically to the necessity of distinguishing between odors, suggesting that it is tied to stimulus salience rather than valence.

To attain a single glomerulus resolution in assessing the selective plasticity of TH+ cells, we stimulated the M72-GFP mice with acetophenone, a ligand of the M72 receptor [45]. This approach allowed us to precisely quantify TH expression surrounding GFP-tagged glomeruli (S2G Fig). Odor discrimination assay but not indiscriminative reward association increased TH counts and density around M72 glomeruli (S2H and S2I Fig). Prolonged odor exposures did not increase the volume of individual M72 glomerulus (S2J Fig). These results suggest that plasticity of TH expression is tied to odor contrast and highlights the role of SACs in refining odor discrimination when precise sensory processing is critical for goal-directed behaviors.

### Discriminative learning differentially affects SAC connection to other neurons

**Fig 3:**
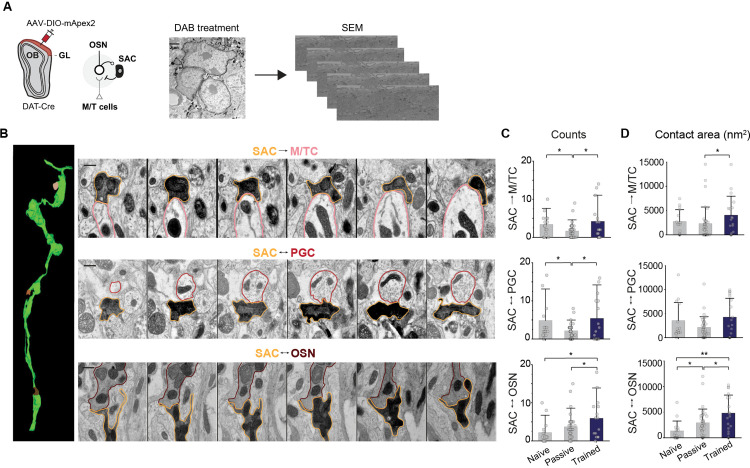
Discriminative learning differentially affects SAC connections to other neurons. (A) Schematic of mAPEX2 expression in SACs followed by DAB staining and serial sections for 3D reconstruction. (B) 3D modeling of the DAB-stained SAC processes. Imaging of SAC interactions in the glomerular layer is shown across multiple serial sections, scale bar: 500 nm. (C, D) Distribution of SAC synaptic contacts **(C)** and the corresponding surface area of each contact **(D)** in OBs of naïve (*n* = 15), passively exposed (*n* = 37), and trained animals (*n* = 18; 2 mice per condition). *P < 0.05, **P < 0.01, Kruskal–Wallis followed by Dunn’s post-hoc test. Data for this figure are provided in S1 Data.

Our observations that TH expression selectively increases in olfactory bulb regions activated by reward-associated odors during discrimination learning confirm that local molecular changes occur with learning. We proposed that this selective increase in TH expression reflects synaptic plasticity within the SACs, contributing to the modulation of glomerular activity. It is difficult to directly assess the synaptic input from the SACs to the OSN axons. To address this problem, we designed an adeno-associated virus (AAV) that carried a Cre-dependent expression cassette that expressed membrane-anchored ascorbic peroxidase (mAPEX2) [46]. The expression of mAPEX2 allowed selective labeling of SAC compartments with biotin, and enabled us to evaluate structural changes associated with the SACs during learning. DAB staining and scanning electron microscopy (SEM) provided a detailed view of synaptic interactions involving SACs within the glomerular layer (Fig 3A).

We classified glomerular processes into 3 known SAC interacting cells, the OSNs, mitral/tufted cells (MTCs), and periglomerular cells (PGCs; Fig 3B). Identification of OSNs, MTCs and PGCs was done using previously described criteria [47] (“Materials and methods”). Analysis of SAC processes across multiple samples showed that passive odor exposure increased the contact between SACs and the OSNs, with a significant change in contact areas (*Z*(2) = 2.3, *p* = 0.017), but there was also significant reduction in the number of contacts between SACs with MTCs and PGCs (Fig 3C and 3D; MTP: *Z*(2) = 2.3, *p* = 0.017; PGC: *Z*(2) = 1.9, *p* = 0.04). In contrast, when odor exposure was paired with reward, we did not observe obvious changes of SAC → PGC and SAC → MTC contacts, but the increase of SAC → OSN was significantly higher than that for passive exposure (*Z*(2) = 2.2, *p* = 0.032). The data suggests that synaptic contacts between the SACs and other cell types are differentially regulated by odor experience. In both cases, odor-driven activity increases the contact between SACs and OSN axons, but in the case of passive exposure, this increase appears compensated by corresponding reduction of contact with PGCs and MTCs, possibly a result of homeostatic regulation. An increase in the SAC → OSN connection will increase the dynamic range of SAC feedback to the OSNs, allowing a more prominent role in gain control.

### Dopaminergic SACs modulate valence-dependent glomerular responses

**Fig 4:**
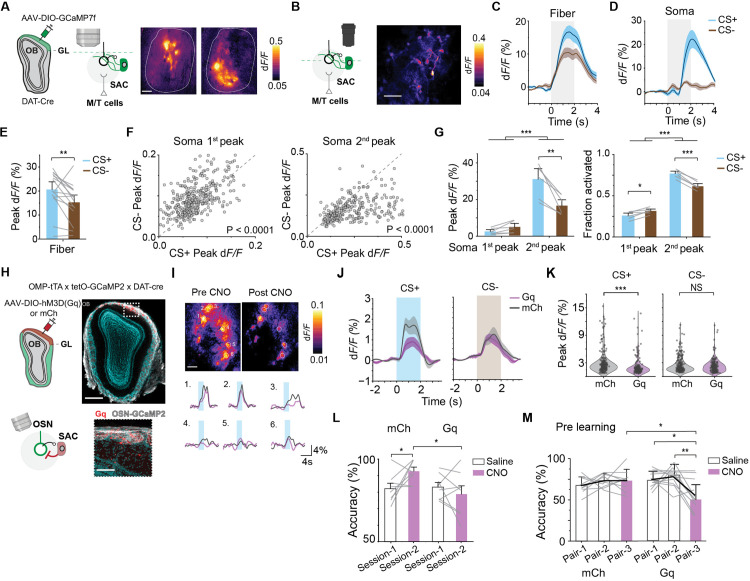
Dopaminergic SACs modulate valence-dependent glomerular responses. (A) SAC dendritic field post expression of GCaMP7f in DAT-Cre mice during odor discrimination, scale bar 200 µm. (B) Representative image showing SAC soma responses in trained animals, scale bar: 50 µm (C, D) Average responses from dendritic field (**C**; n = 15 animals) and soma (**D**; n = 6 animals) of SACs. Shaded areas indicate odor delivery. Data are mean ± standard error. (E) Bar graph shows average peak responses for dendritic fields. **P < 0.01, one-way repeated measures ANOVA followed by Tukey’s *post hoc* test. (F) Peak responses of all somas during the first and second phases across different odor valences. One-way repeated measures ANOVA followed by Tukey’s *post hoc* test. (G) Average peak responses (left) and proportion of activated neurons (right) separated according to the biphasic responses. *P < 0.05, **P < 0.01, ***P < 0.001, two-way repeated measures ANOVA followed by Tukey’s *post hoc* test. (H) Schematics of DREADDq (Gq) expression in SACs of *OMP-tTA; tetO-GCaMP2; DAT-Cre* animals, scale bar: 500 µm. Magnified area shows Gq expression (red) in dopaminergic cells surrounding the glomeruli (white), scale bar: 50 µm. (I) CS+ evoked glomeruli activity pre-and post CNO injection, scale bar 200 µm (grey: pre CNO; purple: post CNO). (J) Average glomerular response to CS+ (left) and CS− (right) odors post CNO injection in mCh (grey, *n* = 8) or Gq (purple, *n* = 8) expressing SACs. (K) Violin plots show comparisons of individual glomerular responses across mCh and Gq animals for CS+ (left) and CS− (right) odors. ***P < 0.001, mixed-design ANOVA followed by Tukey’s *post hoc* test. (L) Accuracy in discrimination of the same odor pair post-Saline (Session-1) and CNO (Session-2) injections in mCh (left, *n* = 8) and Gq (right, *n* = 8) animals. *P < 0.05, mixed-design ANOVA followed by Tukey’s *post hoc* test. (M) Accuracy in odor acquisition post saline (Pair-2) and CNO (Pair-3) injections in mCh (*n* = 9) and Gq (*n* = 11) animals. *P < 0.05, **P < 0.01, mixed-design ANOVA followed by Tukey’s *post hoc* test. Data for this figure are provided in S1 Data.

Molecular and structural changes in the OB indicate that the SACs serve as a key connection between odor input and the reward signals. To investigate the functional consequence of SAC adaptability, we transduced the SACs of *DAT-Cre* mice with an AAV that conditionally expressed GCaMP7f [48,49] and used widefield imaging to record calcium signals from SAC dendritic fields and two-photon imaging to observe SAC somas (Fig 4A and 4B). Odor stimulation activated the SAC dendrites in areas aligned with glomerular activation, indicating that the signals reflected response to odor-evoked activity from the OSN axons. The response to the CS− odor plateaued but those to the CS+ odor continued to rise both at the dendritic and somatic levels (Fig 4C–4E; *q*(14) = 4.37, *p* = 0.008). Response dynamics were similar between two odors in mice passively exposed to both odors (S3A–S3C Fig). While response latency at the dendritic field was similar to those of the glomerular activity, there was significant temporal divergence between CS+ and CS− odor onset in the soma (S3D Fig). Soma responses exhibited a biphasic pattern, with the first peak recruiting a significantly higher proportion of SAC somas for CS− odors (*q*(352) = 13.1, *p* < 0.0001). In the later response phase, CS+ odors elicited stronger responses and recruited more cells than CS− odors (Fig 4F and 4G; second peak: *q*(352) = 15.5, *p* < 0.0001). This divergence suggests that inhibitory mechanisms exert transient control over the soma to shape olfactory coding based on valence. The muted SAC soma response to CS− odors is consistent with a weaker top–down modulation associated with punishment (see below).

To further assess the glomerular layer interactions, we investigated the dendritic responses of the principal projection neurons in the OB, the MTCs (S3E Fig). In contrast to what we observed for the SAC dendrites, MTC dendrites had relatively smaller responses in awake mice that received passive exposure [32,50,51] (S3F Fig). In those with discriminatory training, response to both CS+ and CS− odors increased. Consistent with the SAC dendritic activity, the CS+ odors activated MTC dendritic field significantly more than the CS− odors (S3G Fig).

The SACs form a network interaction between the OSN axons and the MTCs. While its role in modulating the latter is proposed [38,52–54], presynaptic gain control by the SACs is less clear [55,56]. We hypothesize that the SACs exert presynaptic inhibition and selective modulation of the inhibitory strengths differentially modulates odor activity and enhances discrimination. To test this hypothesis, we injected AAVs expressing excitatory DREADD receptors hM3D(Gq) together with GCaMP7f in the OB of DAT-Cre mice [57] (S4A Fig). CNO injections significantly suppressed odor driven responses in SAC dendrites compared to saline conditions (S4B and S4C Fig). This observation showed that the chemogenetic activation of SACs shifts the olfactory circuitry towards a more inhibited state [58]. To test whether this broad activation of the SACs can selectively modulate OSN axon and tie them to behavioral phenotypes in odor training, we generated *OMP-tTA; tetO-GCaMP2; DAT-Cre* mouse line for imaging glomerular responses and expressed Gq virus in the SACs (Figs 4H–4I and S4D–S4E). While CS− odor responses were not affected by SAC excitation, CS+ responses were diminished (Fig 4J and 4K; CS+: *q*(436) = 6.44, *p* < 0.0001; CS−: *q*(436) = 1.52, *p* = 0.7). We observed a broad decrease in responsiveness of glomeruli across the dorsal surface (S4F Fig). This result indicated that SACs inhibited OSN axon activity. To examine the role of the SACs in odor discrimination, we trained mice to discriminate two odors in consecutive training sessions. CNO injection in the second training session did not disrupt improvement of accuracy in control mice injected with an AAV expressing mCherry but prevented performance improvement in those injected with Gq (Fig 4L; 14% reduction in accuracy in Gq animals compared to mCh animals post CNO injections *q*(14) = 3.96, *p* = 0.014). Additionally, we trained the AAV injected animals to discriminate new pairs of odors with or without CNO injections. Animals injected with saline continued to learn to discriminate new odor pairs, but CNO injection abolished this learning (Fig 4M; pair-2 versus pair-3 (Gq): *q*(36) = 6.08, *p* = 0.0016). Thus, direct activation of the SACs overrode learning associated changes that differentially modulated glomerular responses and prevented further improvement in odor discrimination.

To confirm the necessity of SACs in establishing new odor discrimination, we injected inhibitory DREADD receptors hM4D(Gi) in the OB of DAT-Cre animals. Imaging SAC dendrites using calcium indicators revealed CNO injection enhanced odor induced activity (S4G–S4I Fig). This result is consistent with SAC’s role in providing inhibitory modulation of glomeruli activity. In behavioral tests, mice transduced with the control AAVs successfully discriminated new odor pairs post CNO injection. In contrast, mice injected with the Gi failed to discriminate new odor pairs when SAC activity was silenced by CNO (S4J and S4K Fig). Taken together, these results indicate that the SACs control odor selection in the glomeruli through a presynaptic mechanism and are essential for odor discrimination.

### Top–down cholinergic input modulates glomerular responses and controls olfactory discrimination

**Fig 5:**
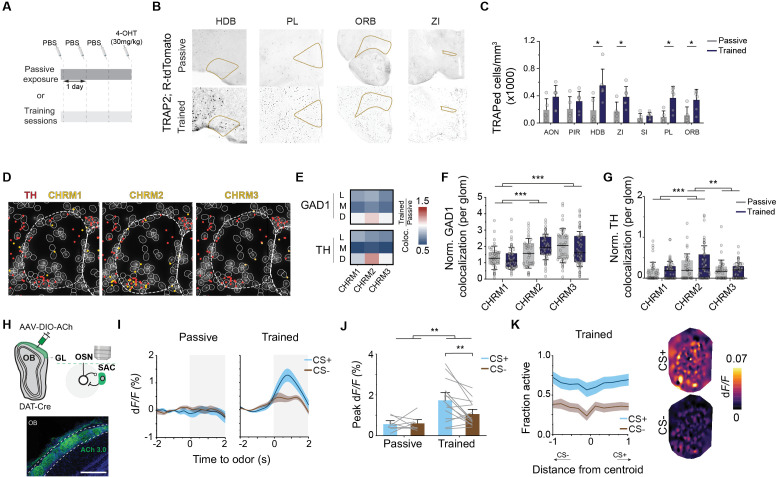
Top–down cholinergic input mediates selective modulation of reward odors. (A) Strategy to identify tdTomato cells in the brain during passive odor exposure (top) or post odor association (bottom) in *TRAP2; Ai14* animals. (B) Example brain sections from *TRAP2; Ai14* passively exposed (top) and trained (bottom) animals. Trapped cells are shown in black, yellow lines define quantified areas. (C) Quantification of tdTomato+ cell density in brain areas after passive odor exposure (*n* = 5, Passive, dark grey) or odor value association (*n* = 6, Trained, light grey), *P < 0.05, Wilcoxon rank-sum test (two-tailed). (D) Co-localization of TH and neuromodulatory receptor transcripts in the glomerular layer. (E) Change in colocalization between interneurons and receptor transcripts as a proportion of all cells. Red color indicates an increase in colocalization between genes with training compared to passive odor exposure. (F**–**G) Bar plots show normalized colocalization counts of muscarinic cholinergic receptors with GAD1 **(F)** and TH transcripts **(G)** per glomerulus. **P < 0.01, ***P < 0.001, two-way ANOVA followed by Tukey’s *post hoc* test. (H) Paradigm for imaging ACh transients during odor discrimination. Example OB section shows ACh GRAB sensor expression by the SACs in DAT-Cre mice, scale bar 200 µm. (I) Average activity trace for all recorded ROIs in passively exposed (left, *n* = 8) and trained (right, *n* = 15) animals. (J) Bar graph shows average peak responses. **P < 0.01, mixed-design ANOVA followed by Tukey’s *post hoc* test. (K) Left panel: Fraction of responsive ROI across the dorsal OB. Right panel: Heatmaps show spatial distribution of peak ACh responses for CS+ and CS− odors after training. Data for this figure are provided in S1 Data.

Perceptual decision-making is achieved through a coordinated effort across cortical and subcortical brain regions and their interactions with sensory areas. We examined brain-wide activities using *Trap2; Ai14* mice that underwent discriminative learning or passive odor exposure to mark activated cells [59] (Fig 5A). We found an overall increase of activated cells within subregions of the thalamus and isocortex, which play crucial roles in integration of sensory inputs to orchestrate appropriate behavioral responses (S5A Fig). Additionally, brain areas known to mediate associative learning, including zona incerta, prelimbic area, and the orbitofrontal cortex were enriched with tdTomato positive cells in trained animals (Fig 5B). Notably, the horizontal limb of the diagonal band (HDB) was the only region that directly innervates the OB with a significant increase in active cells (Fig 5C; *Z* = 2.28, *p* = 0.017).

We hypothesize that learning associated cholinergic activity serves as a relevant signal to modulate early sensory responses. *Ex-vivo* studies indicated an inhibitory drive of acetylcholine (ACh) onto the SACs [60], which has been confirmed with our in vivo studies [58]. Spatial transcriptomic analysis confirmed the localization of muscarinic cholinergic receptors in the glomerular layer, with type II muscarinic receptors (CHRM2) showing a significantly higher colocalization with TH (Fig 5D–5G; CHRM2 versus CHRM1: *q*(283) = 5.4, *p* = 0.0004; CHRM2 versus CHRM3: *q*(283) = 4.9, *p* = 0.0016).

To verify that HDB cholinergic input directly acts on the SACs, we used AAV9 to express the ACh GRAB sensor in *DAT-Cre* mice and recorded ACh transients [61] (Fig 5H). In passively exposed animals, odor stimulation did not elicit notable ACh release onto the SACs. In trained animals, cholinergic activity was elicited by both CS+ and CS− odors but the activity level was dependent on the valence of the stimuli; response to CS+ odor was significantly higher than CS− (Fig 5I–5J; *q*(21) = 5.69, *p* = 0.003). Cholinergic activity was observed across the OB surface without apparent spatial selectivity (Fig 5K). Thus, ACh release specificity is tied to odor valence, not its identity.

**Fig 6:**
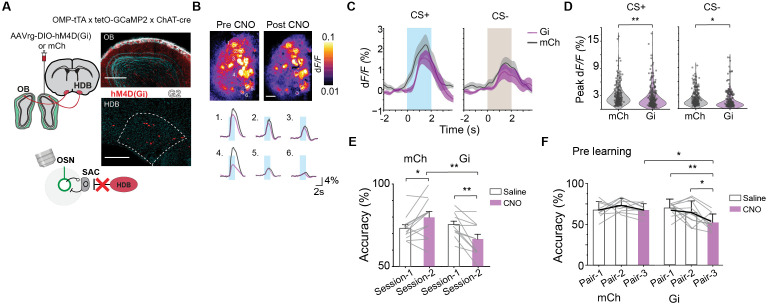
Top–down cholinergic input modulates glomerular responses and controls olfactory discrimination. (A) Schematics of DREADDi (Gi) expression in cholinergic HDB neurons of *OMP-tTA; tetO-GCaMP2; ChAT-Cre* animals. Example injection site shows Gi expression (red) in cholinergic cells in HDB (bottom) and projections in OB (top), scale bar: 200 µm. (B) CS+ evoked glomeruli activity pre-and post CNO injection, scale bar 200 µm (grey: pre CNO; purple: post CNO). (C) Average glomerular response to CS+ (left) and CS− (right) odors post CNO injection in mCh (grey, *n* = 6) or Gi (purple, *n* = 7) expressing cholinergic cells. (D) Violin plots show comparison of individual glomerular responses across mCh and Gi animals for CS+ (left) and CS− (right) odors. *P < 0.05, **P < 0.01, mixed-design ANOVA followed by Tukey’s *post hoc* test. (E) Accuracy in discriminating the same odor pair post-Saline (Session-1) and CNO (Session-2) injections in mCh (left, *n* = 12) and Gi (right, *n* = 12) animals. *P < 0.05, **P < 0.01, mixed-design ANOVA followed by Tukey’s post hoc test. (F) Accuracy in odor acquisition post saline (Pair-2) and CNO (Pair-3) injections in mCh (*n* = 9) and Gi (*n* = 11) animals. *P < 0.05, **P < 0.01, mixed-design ANOVA followed by Tukey’s *post hoc* test. Data for this figure are provided in S1 Data.

The lack of spatial pattern of ACh release for a given odor suggests that specific modulation of glomerular activity must arise from the SACs as a result of discriminative learning. We hypothesize that ACh produces a stronger disinhibition to glomeruli activated by reward odor by inhibiting the SACs. To test this hypothesis, we crossed the *OMP-tTA; tetO-GCaMP2* mice with *ChAT-Cre* line and injected retrograde-AAV in the OB to express Gi to mark the long-range cholinergic projections from the HDB (Fig 6A and S5B). We monitored calcium activity of the OSNs with GCaMP2 in trained Gi expressing animals injected with CNO (Fig 6B). CNO induced silencing of cholinergic input significantly reduced activity for both odors, but reduction was more prominent for the CS+ odors (Figs 6C–6D and S5C; CS+: *q*(528) = 4.57, *p* = 0.0071; CS−: *q*(528) = 4.11, *p* = 0.019). This result was consistent with the hypothesis that cholinergic input inhibited the SACs. In the absence of this inhibition, SACs continued to inhibit the glomeruli.

We then tested the role of cholinergic projections in odor discrimination assay. Animals transduced with control AAVs continued to increase discrimination accuracy during training with CNO injection (*q*(22) = 3.62, *p* = 0.017). However, CNO injection in Gi expressing animals reduced previously acquired discrimination accuracy (Fig 6E; 9% reduction in accuracy in Gi animals post CNO injections *q*(22) = 4.75, *p* = 0.0028). Similar observations were made when Gi was expressed directly in the HDB (S5D Fig). Moreover, inhibition of cholinergic projections prevented learning of new odor associations (Fig 6F; pair-2 versus pair-3 (Gi): *q*(36) = 4.49, *p* = 0.035). Thus, cholinergic input to the OB is required to maintain discrimination as well as learn new odor-value associations.

### Recapitulating cholinergic engagement with chemogenetic manipulation

**Fig 7:**
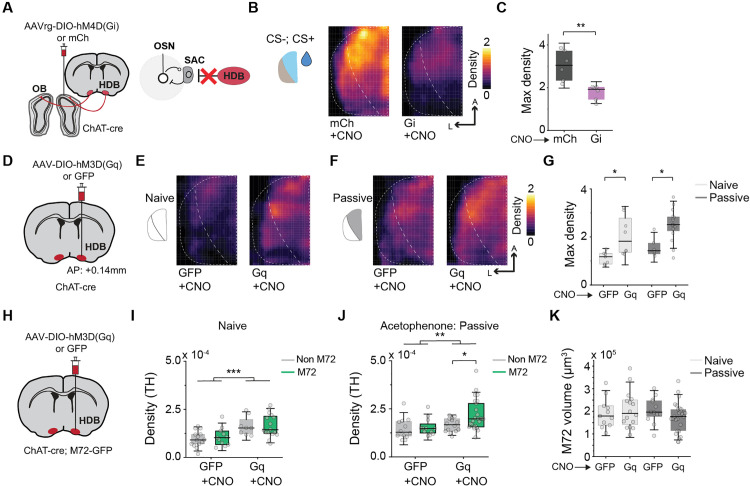
Recapitulating cholinergic engagement with chemogenetic manipulation. (A) Strategy to manipulate HDB projections during odor training using DREADDi (Gi) expression in ChAT-Cre animals. (B, C) Spatial density of TH immunofluorescence over the dorsal OB after odor training in mCh (left) and Gi (right) animals. **P < 0.01, two sample *t* test (two-tailed). (D) Strategy to recruit cholinergic HDB activity using DREADDq (Gq) expression. (E, F) Spatial density of TH immunofluorescence following recruitment of HDB activity in *ChAT-Cre* animals using Gq without odor exposure **(E)** and post passive exposure (F). (G) Box plots show comparison of max TH density over dorsal OB in GFP and Gq injected animals across odor conditions. *P < 0.05, two-way ANOVA followed by Tukey’s *post hoc* test. (H) Strategy to recruit cholinergic HDB activity in *ChAT-Cre; M72-GFP; Ai14* animals using Gq. (I, J) Density of TH expression surrounding M72-GFP glomeruli compared with antero-dorsal (non-M72) glomeruli without odor exposure **(I)** and post passive exposure (J). *P < 0.05, **P < 0.01, ***P < 0.001, two-way ANOVA followed by Tukey’s *post hoc* test. (K) Volume of each M72 glomerulus following odor conditions with (Gq) and without (GFP) cholinergic recruitment. Data for this figure are provided in S1 Data.

The evidence so far indicates that cholinergic input from the basal forebrain provides the signal to the SACs to induce both molecular and functional changes that enable enhanced odor discrimination. We sought to test the hypothesis by directly manipulating cholinergic activity through chemogenetic means. We injected retro AAVs that conditionally express hM4D(Gi) or mCherry into the bulb of *ChAT-Cre* animals and examined the expression of TH following discriminatory training with CNO injection (Fig 7A). Suppression of cholinergic input to the OB was sufficient to suppress training induced TH expression in glomerular region corresponding to both CS+ and CS− odors (Fig 7B and 7C; *t*(10) = 3.97, *p* = 0.0026). Post-training TH suppression was also evident when we inhibited the cholinergic cells by injected Gi directly in HDB (S5E Fig).

In a converse experiment, we injected excitatory DREADDs or control AAVs in HDB of *ChAT-Cre* animals (Fig 7D). In naïve animals, cholinergic activation significantly increased overall TH expression without an odor experience (Fig 7E; *q*(36) = 4.13, *p* = 0.029). Concurrent activation of cholinergic input in Gq animals that underwent passive odor exposure enhanced TH expression, recapitulating the effect of CS+ training (Fig 7F and 7G; *q*(36) = 4.55, *p* = 0.014). To get a finer grain examination of this effect, we used a *ChAT-Cre; M72-GFP; Ai14* mouse line to examine TH expression around the M72 glomerulus (Fig 7H). When no external odor was provided, activation of cholinergic cells in HDB using CNO led to increased TH expression across the glomeruli (Fig 7I; *q*(48) = 6.21, *p* < 0.0001). Providing acetophenone passively induced a slight increase in TH expression around the M72 glomeruli, but Gq activation led to a significantly higher TH expression (Fig 7J; *q*(63) = 4, *p* < 0.031). There was no overall change in the volume of the M72 glomeruli across control or Gq groups with or without odor experience (Fig 7K).

## Discussion

Sensory inputs are translated into patterns of neuronal activity throughout the processing stages. Each stimulus activates a group of neurons that encodes its identity. Neural patterns are used to differentiate between the stimuli to drive specific behavioral and motor actions. Association of these sensory patterns with behavioral consequences enables the animals to make decisions that maximize reward or avoid punishment. These associations establish a foundation for sensory-guided behaviors. Whereas the establishment of these associations has been extensively studied, it is not clear how the learning process leads to changes in sensory coding to enhance discrimination. Individual neurons often participate in the encoding of multiple stimuli, creating overlapping patterns. Our study demonstrates that discriminative learning enhances the salience of sensory signals to improve the brain’s ability to distinguish patterns of neuronal activity and drive distinctive responses. We further reveal the neural circuit and molecular changes underlying discriminative learning in the early processing stages of mammalian olfactory system.

**Fig 8:**
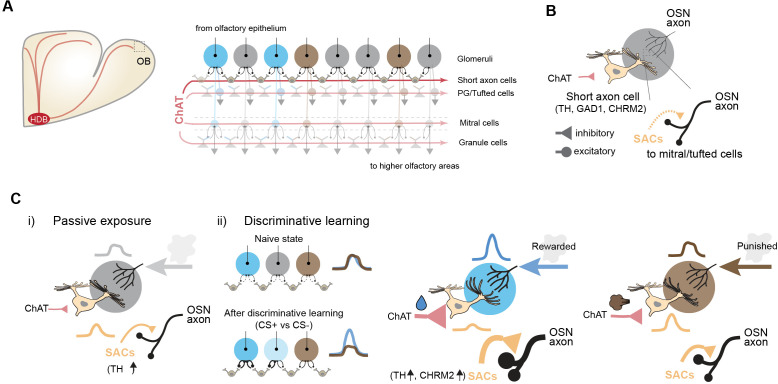
Cholinergic modulation of olfactory bulb circuits during odor exposure and discriminative learning. (A) Schematic of basal forebrain cholinergic projections from the HDB to the OB. In the right panel, major cell types are labeled. Within the glomerular layer, excitatory OSN axons innervate the MTCs, as well as inhibitory SACs and granule cells. Cholinergic input (ChAT-positive; maroon) innervates multiple cell types. Cholinergic input to the SACs is highlighted. (B) Circuit diagram showing SAC-mediated inhibition of OSN input. SACs express tyrosine hydroxylase (TH), glutamate decarboxylase 1 (GAD1), and muscarinic receptor CHRM2. Cholinergic input from the HDB acts on SACs to regulate inhibition of sensory input. (C) Cholinergic–SAC interactions under different learning conditions. (i) Passive exposure: Cholinergic input modestly recruits SAC activity, with increased TH expression, broadly modulating OSN input. (ii) Discriminative learning: In the naïve state, SAC connectivity and cholinergic modulation are uniform across glomeruli. After training (CS+ vs. CS− discrimination), SAC molecular markers (TH, CHRM2) are upregulated selectively in CS+ associated glomeruli, enhancing cholinergic modulation and reciprocal inhibition to the OSNs. For rewarded odor, cholinergic input strongly inhibits SACs and disinhibits OSN–MTC pathways, amplifying CS+ responses. In the punished condition, SAC-mediated inhibition is maintained for CS− glomeruli, suppressing responses.

Discriminative learning involves top–down inputs, which include feedback from areas involved in attention, expectation, or previous experiences to modulate how sensory information is processed. These inputs serve as teaching signals to enhance pattern separation. The olfactory bulb participates in several feedback and feedforward loops, including neuromodulatory input from the locus coeruleus (noradrenaline), the raphe nuclei (serotonin), and the basal forebrain (acetylcholine) [62–64]. Previous studies have established that cholinergic input from the basal forebrain modulates olfactory bulb activity to enhance odor detection, reduce habituation, and facilitate discrimination even in the absence of explicit reward or punishment associations [25,26,65–67]. Additionally, the strength of cholinergic activity in the basal forebrain has been linked to odor valence [68,69]. While these studies proposed that acetylcholine influences bulbar output that may facilitate its role in discrimination, the specific circuit-level mechanisms by which cholinergic input shapes odor processing have remained unclear (Fig 8A).

How does cholinergic input enhance odor discrimination? Pattern separation essentially involves strengthening the representation of distinctive features of each stimulus while minimizing the overlap between patterns representing different stimuli, allowing the brain to store and recall memories more accurately and to make more precise sensory discriminations. A common mechanism for pattern separation is lateral inhibition, which involves inhibitory neurons that suppress the activity of neighboring neurons. The olfactory bulb has a large population of interneurons in both the glomerular and the inner plexiform layers with extensive molecular and morphological heterogeneity [70–73]. Although any type of interneuron could mediate lateral inhibition, our study addresses this gap by identifying dopaminergic SACs as a specific and functionally relevant target of cholinergic input in the glomerular layer (Fig 8B). We demonstrate that SACs are both necessary and sufficient to mediate behavioral enhancement during odor discrimination, and that the selective modulation is shaped by learning. The SACs receive direct input from the OSNs and provide reciprocal inhibition via both dopamine and GABA to the OSNs [55,56]. While our study focused on TH as a selective marker of SAC plasticity, GABAergic transmission likely contributes to presynaptic inhibition of OSNs as well. Dopamine and GABA are expected to act synergistically at the presynaptic site to suppress OSN output. However, our spatial transcriptomic data revealed a learning-dependent increase in TH but not GAD1, suggesting that plasticity is selectively engaged in dopaminergic signaling, and all inhibitory pathways are not equally modulated by experience. Odor evoked activity increases the contact between the SACs and the OSN axons (Fig 8Ci). These changes enable a stronger inhibitory drive onto OSNs, allowing a broader dynamic range for sensory gain control through disinhibition.

We show that top–down cholinergic input is important for regulating these differential gene expression patterns. Critically, we show that at a given trial, the cholinergic input to the olfactory bulb did not exhibit spatial selectivity tied to either odor. Once learned, however, cholinergic input selectively enhances response to the CS+ odor. Notably, this effect is evident in glomeruli tuned to both CS+ and CS− stimuli, which become increasingly selective for the CS+ odor following training (Fig 8Cii). This suggests that reward-driven plasticity sharpens odor representations even within overlapping sensory channels. This preferential enhancement does not reflect a spatial bias in the cholinergic signal itself but rather arises from learning-dependent strengthening of SAC–glomerular connections. One possibility is that SAC-to-OSN inhibition is weak at baseline and becomes functionally relevant only after reward training, leading to selective disinhibition of glomeruli during reward-triggered cholinergic input. Supporting this, we observe increased TH expression near CS+ responsive regions, indicating that synaptic plasticity shapes SAC output to enable spatially biased disinhibition even under diffuse cholinergic input. This spatial specificity also explains why the activation of SACs reduces responses to CS+ odors but has minimal effect on CS− odors. While SAC activation may globally modulate inhibitory tone, its strongest effects are localized to regions where learning has enhanced SAC–OSN connectivity.

While the circuit mechanism we highlight is most prominent in valence-based learning, it also likely contributes to more general forms of sensory plasticity. Indeed, we show that SAC modulation occurred not only during reward-driven tasks but also during passive or non-discriminative exposures. Discriminative learning elevated TH expression in the glomeruli representing CS− odors to levels that were comparable to conditions of non-discriminative reward pairing. The net effect is not only a contrast between CS+ and CS− glomeruli but also an overall increase in TH expression. Given that discriminatory learning occurs within a short timeframe (approximately 30 min) and that neurogenesis typically unfolds over weeks [29,74,75], we speculate that increased TH expression in existing SACs supports enhanced odor discrimination rather than recruiting new SACs. Beyond strengthening SAC–OSN interactions, our data also reveal that odor experience differentially regulates SAC synaptic contacts with other cell types to maintain network stability. During passive odor exposure, increases in SAC input to OSNs are accompanied by a reduction in SAC contacts onto PGCs and MTCs. Since SACs normally inhibit PG cells, a decrease in SAC → PGC connectivity would disinhibit PGCs, allowing them to provide stronger and faster inhibition onto the glomeruli. Concurrently, SACs provide excitatory input onto MTCs via D1 receptor signaling. Thus, a reduction in SAC → MTC connectivity would counteract the heightened odor-evoked activity, preventing excessive excitation of OB output neurons. Together, these homeostatic adjustments enable the olfactory bulb to accommodate increased sensory drive while maintaining balanced output, supporting robust pattern separation during learning.

We show that discriminative learning leads to modulation of sensory responses at the earliest odor processing stage through disinhibition of a special type of interneuron. In addition to SACs, several other OB cell types express cholinergic receptors and may contribute to the modulatory effects we observe (Fig. 8A). PGCs, MTCs and granule cells have been shown to express muscarinic and nicotinic receptor subtypes [64,76]. Activation of muscarinic receptors on PGCs can enhance their inhibitory control over OSN–MTC transmission, potentially sharpening glomerular tuning, whereas nicotinic receptor activation on mitral cells can directly increase their excitability and output gain. Granule cells, which form reciprocal dendrodendritic synapses with MTCs, can also be modulated by muscarinic signaling to adjust lateral inhibition across mitral cell populations [77,78]. Although these pathways were not the primary focus of our study, their recruitment during cholinergic release could act in parallel with SAC-mediated disinhibition, influencing the net outcome on odor discrimination. One limitation of our study is that, in order to clearly identify molecular changes associated with learning, we chose odor pairs that evoke largely non-overlapping glomerular patterns. While this design enabled us to attribute changes unambiguously to either CS+ or CS− glomeruli, it may underestimate the role of SACs in discriminating odors with similar and overlapping patterns. For such odors, SACs may carry specific modulations based on differential dendritic activity. It will be interesting to test this hypothesis using more refined and coordinated imaging studies. Disinhibition at the first stage is likely advantageous in reducing cognitive load at the higher cortical region for rapid decision-making. Importantly, cued attention activates cholinergic input to the SACs and selectively enhances speed and amplitude of glomerular responses for CS+ odors [58]. It is likely that the association between the cue and potential reward elicits predictive cholinergic activity, which provides transient modulation of sensory responses. As learning induced changes permit faster and more accurate decoding of sensory input, the predictive power of the cue diminishes, and the cholinergic activity is disengaged [58]. Together, these studies position SACs as integrators of transient attentional inputs and long-term learning-driven plasticity, enabling refined sensory discrimination. Although most cortical areas do not contain dopaminergic interneurons, the top–down modulation through a disinhibitory circuit can nonetheless offer a general principle of discriminative learning.

## Materials and methods

### Ethics statement

All animal procedures were conducted in accordance with institutional and national guidelines for the care and use of laboratory animals, adhering to the NIH Guide for Care and Use of Animals. The experimental protocols were approved by the Institutional Animal Care and Use Committee at Stowers Institute for medical research under protocol number 2022-151.

### Animals

The mouse strains used include *OMP-tTA* (Jackson Laboratory, Stock no. 017754), *tetO-GCaMP2* (Stock no. 017755), *Chat-Cre* (Stock no. 006410), *DAT-Cre* (Stock no. 006660), *Fos2A-iCreERT2* (TRAP2, Stock no. 030323), *R-tdTomato* (Ai14, Stock no. 007908), *M72-GFP* (Stock no. 006678) and *Cdhr1-Cre* (MMRRC, Stock no. 030952-UCD), previously described in the literature. Both male and female mice, aged 8–10 weeks, were maintained under a 12:12 h reversed light cycle with ad libitum access to food and water until three days before behavioral training. Behavioral and functional imaging experiments were performed during the animals’ dark cycle.

### Odor delivery with olfactometer

Odor delivery was managed using an automated olfactometer, which operated with custom software developed in National Instrument Labview [79]. The specific odorants used are detailed in S1 Table. Each single compound chemical was freshly prepared in mineral oil at a concentration of 3:10^3^ (v/v). These odorants were further diluted in carrier air, maintaining a total flow rate of 400 mL/min for all calcium imaging and behavioral experiments. The diluted odors were delivered directly to the animal’s nose.

### Animal behavior

Mice were subjected to water restriction for three days prior to the start of behavioral training, ensuring they maintained more than 85% of their free drinking weight throughout the experiment. Initially, the mice were acclimated to a head-fixation setup and trained to lick a water port connected to a lick circuit to detect contacts and receive water before any odor exposure. During the pre-training phase, mice were exposed to only the CS+ odor paired with a water reward. Once the mice reliably collected water in over 80% of the trials, they proceeded to the full discrimination training paradigm. The discrimination training involved a pseudorandom sequence of CS+ and CS− odor presentations, totaling 70–80 trials per session. Each trial was separated by an inter-trial interval ranging from 15 to 40 s. Following a 0.3-s delay after odor delivery, a response window of 3 s was provided. Licks within this window for the CS+ odor resulted in a reward of 7.5 µL of water, while licks for the CS− odor were punished with a brief 2-s air puff. Trials without a response were recorded as a miss for CS+ or a correct rejection for CS−. Discrimination accuracy was calculated by averaging responses for CS+ trials and withholdings for CS− trials within the response window. Behavioral training and odor exposure experiments for all M72-GFP mice were done in a free moving setup and lasted for a total of 7 days. The airflow from olfactometer was directed to a port opening in an experiment box with dimensions of 27 × 7 × 8 cm (length × width × height). IR break events were registered and triggered a brief noise (CS−) and/or water (CS+) depending on the paradigm. Imaging and fluorescent data were analyzed from mice that achieved a discrimination accuracy above 70% in behavioral experiments, ensuring that the findings reflect genuine cognitive engagement and accurate odor discrimination. For non-discriminatory pairings, IR break events for both odors triggered water. For passive exposure, mice were exposed to the odor without any valence association for a total of 7 days.

### Stereotaxic surgeries

Mice were anesthetized with an intraperitoneal injection of a ketamine/xylazine cocktail (100 mg/kg and 10 mg/kg, respectively) and a subcutaneous injection of buprenorphine (0.1 mg/kg) prior to being placed in a stereotactic frame. Body temperature was maintained at 37 °C using a heating pad. Viruses were injected at a final titer of 0.5–1 × 10^13^ vg/ml. For the olfactory bulb experiments, four small cavities (two on each olfactory bulb, both anterior and posterior) were created using a dental drill. At each injection site, 250–350 nL of the virus solution was injected at multiple depths, spanning 80–150 μm below the skull surface. Three to four weeks after the virus injections, the animals were anesthetized again for cranial window preparation over the olfactory bulbs. For widefield glomeruli, SAC, and MT dendritic field imaging, the skull above both olfactory bulbs was thinned using a dental drill, and a custom-made headbar was secured to the skull with dental cement. Following surgery, the mice received buprenorphine for the next two days and were allowed to recover for at least one week before undergoing imaging experiments.

### Head-fixed imaging

For calcium imaging of glomeruli, the generation of the GCaMP2 mice has been previously described [31]. To image dendritic field responses and acetylcholine transients, AAV1-Syn-FLEX-jGCaMP7f (Addgene, 104492-AAV1), or AAV9-hSyn-DIO-Ach3 (WZ biosciences) respectively was bilaterally expressed in OBs of *Cdhr1-Cre* and *DAT-Cre* animals. Images were collected using an Olympus BX60WI microscope using 4× air lens (Olympus XLFLUOR4X/340, NA 0.28). The images were captured at a resolution of 512 × 512 with a 2 × 2 binning, and the sampling rate was set at 10 Hz for the SAC DREADD and ACh GRAB experiments and 13.3 Hz otherwise. For capturing the activity of SAC somas, AAV1-Syn-FLEX-jGCaMP7f was introduced into OBs of DAT-cre animals at a depth of 100–200 µm from surface. Imaging was performed using an Olympus 2-photon microscope (FLUOVIEW FVMPE-RS), utilizing a 940 nm excitation laser and a 25× water immersion lens (Olympus XLPLN25XWMP, NA 1.05). Images were collected with a resonant scanner equipped with a GaAsP detector, at a resolution of 512 × 512 and a sampling rate of 15 Hz.

### Histology

Mice were euthanized following anesthesia with urethane and perfused with 4% paraformaldehyde (PFA) in phosphate-buffered saline (PBS). The brains were then incubated in 4% PFA overnight at 4 °C. Coronal sections (60 µm thick) were cut using a vibratome (Leica VT1000S). Primary antibodies were prepared at a 1:1000 dilution for Chicken anti-GFP (Abcam, ab13970), Rabbit anti-RFP (Rockland, 600-401-379), Goat anti-tdT (Origene, AB8181-200), and Rabbit anti-cFos (Abcam, ab190289) or at a 1:400 dilution for Rabbit anti-TH (MilliporeSigma, ab152) and Goat anti-ChAT (MilliporeSigma, ab144p). These antibodies were diluted with a solution containing 1:20 Donkey serum, 1:20 DMSO, and PBST (0.1% Triton X-100 in PBS) and applied to the sections for overnight incubation at room temperature. After primary antibody incubation, the sections were washed three times for 30 min each with PBST. Secondary antibodies were then applied at a 1:1000 dilution, including Donkey anti-Chicken 488 (Invitrogen, A78948), Donkey anti-Rabbit 568 (Invitrogen, A10042), Donkey anti-Rabbit 647 (Invitrogen, A32795), Donkey anti-Goat 568 (Invitrogen, A11057), and Donkey anti-Goat 647 (Invitrogen, A21447). The secondary antibody solution also contained 1:20 Donkey serum, 1:20 DMSO, and 1:1000 DAPI (1 µg/ml, MilliporeSigma, 268298), and the sections were incubated overnight at room temperature. Images were acquired using an Olympus VS120 Virtual Slide Microscope at 10× magnification with 2 × 2 binning for TH expression quantifications and to confirm the location and expression levels of jGCaMP7f, Ach3.0, GFP, mCherry, and DREADDs.

### Multiplex smFISH

Unfixed brain samples from passively exposed and trained animals were submerged in O.C.T. and snap-frozen for cryosectioning (10 µm) onto slides and sent to Resolve Biosciences for processing on the Molecular Cartography platform. 100 genes were probed for the OB samples (S2 Table). Following sample imaging, cell segmentation was performed in QuPath, and further analysis was performed in R programming language using Seurat package. Briefly, all samples were merged from various experimental, normalized, and variable features identified. PCA facilitated dimensionality reduction, first 15 principal components were used for identification of cellular neighbors followed by clustering and visualization through UMAP. Gene marker detection was conducted with a Wilcoxon Rank Sum test. For glomerular resolution, individual glomeruli were manually traced and transcripts within the ROIs recorded using ImageJ’s polylux plugin. Blinding was maintained throughout the tissue treatment and initial processing phases for smFISH experiments. Preprocessing steps including cell and glomeruli segmentation were performed on anonymized images using Seurat and ImageJ to avoid any bias in data collection. Colocalization between genes was computed using pixel-wise spatial proximity measures. For each image, a colocalization value was calculated based on the number of Gene2 pixels found within a fixed spatial radius of each Gene1 pixel (allowing one-to-many pixel associations). To correct for variations in total expression between conditions, the raw colocalization value was normalized by the geometric mean of the total number of Gene1+ and Gene2+ pixels in the same region.

### TH density quantification

For quantifying TH expression around the M72 glomerulus, images were collected using a Nikon AT-AT spinning disk confocal microscope at 40× resolution. TH+ somas were manually counted within the imaging volume encompassing the M72 glomerulus and its immediate surroundings. Density was calculated from counts of TH-labelled somata across a *z*-stack encompassing the entire depth of GFP labelled M72 glomerulus spanning 2–3 serial sections. Volume of M72 glomeruli was calculated by modeling each glomerulus as cojoined frustums. Measurements for the diameters of the frustum bases and their heights were extracted manually using ImageJ based on the maximum cross section of M72 glomerulus observed in the *z*-stack. For OB wide quantifications of TH expression, serial sections were collected maintaining the anterior posterior axis and images were acquired using an Olympus VS120 Virtual Slide Microscope at 10× magnification without binning in preprocessing steps. Density was calculated by binning the cell counts from automated cell segmentation in QuPath into manually drawn dorsal glomerular layer areas to obtain a single vector of expression per OB slice. Expression bias was calculated by subtracting the mean TH expression in the CS+ zone from that in the CS− zone and then normalizing this difference by the standard deviation of TH expression across all zones. For TH density measurements, experimenters were blinded during the segmentation of dorsal areas in QuPath and subsequent cell counting where consistent thresholding parameters were applied across all samples.

### mAPEX plasmid construction

For the AAV-DIO-mAPEX2 construct, the mAPEX2 coding region was subcloned from the plasmid that contains membrane localization signal of MRRTKQVEKNDEDQKI with APEX2 coding region from pcDNA3 APEX2 NES plasmid (Addgene #49386). mRFP coding region was cloned from plasmid pShuttle-mRFP-gsk3 s9a (Addgene #24371). The fragments were assembled into AscI and BmtI sites of the plasmid AAV-hsyn-DIO-EGFP (Addgene Plasmid #50457) using Gibson assembly (NEB #E2611) to generate the AAV-hsyn-DIO-mAPEX2-T2A-RFP construct. The purified construct was packaged into AAV9 virus at WZbioscience.

### DAB staining and SEM

*DAT-Cre* animals were injected with AAVs carrying membrane signal tagged Apex2 at 4 weeks old. Post treatment, mice were perfused with 2% PFA and 2.5% glutaraldehyde solution. The brains were collected and stored in the same solution for 1 hr at 4 °C. Brain slices were cut by a vibratome and then subjected to DAB staining. After the DAB reaction, the tissue slices were washed with cacodylate buffer, further fixed in 2.5% glutaraldehyde for 2 hrs to stabilize the DAB reaction product. Afterwards, the tissues were postfixed with 1% Osmium for 1 hr, washed, stained with 0.5% uranyl acetate overnight. The tissues were then dehydrated with a serial gradient of ethanol and infiltrated into Epon resin. Ultrathin sections were cut on an ultramicrotome (UC6, Leica) and imaged using a Tecnai Biotwin electron microscopy operated at 80 kV. For SEM array tomography serial sections were cut at 80 nm thickness on a Leica UC7 ultramicrotome and dried down onto a slide placed into a Diatome jumbo diamond knife. The sections were then post stained with 4% uranyl acetate in 70% methanol and Sato’s triple lead stain for 6 min each, and the slide coated with 4nm carbon in a Leica ACE600 coater [80]. Imaging was performed in a Zeiss Merlin SEM using a BSD4 detector at 8–10 kV and 700 pA with Atlas 5 software. After image acquisition, stacks were registered with the IMOD program and analyzed using napari [81]. Dorsal OB sections were chosen following standardized quality checks necessary for SEM preparation, resulting in random sampling of dorsal sections rather than a specific focus on particular glomeruli. Identification of SAC processes was based on increased electron density resulting from DAB treatment providing strong contrast enhancement specific to SACs. Putative contacts were defined as membrane juxtapositions between SAC processes and their postsynaptic targets with no visible extracellular space. Individual cell types were first outlined by hand using the Napari image viewer. We annotated contacts across multiple serial sections and confirmed that they were consistently observed across at least 2–3 consecutive slices to reduce false positives. OSNs were distinguished by their darker cytoplasm and asymmetric synaptic contacts. MTCs were pale and had scattered clusters of spherical vesicles. PGCs were densely packed with large, flattened vesicles and synapsed with symmetrical thickening.

### DREADD based activation and inhibition using CNO application

Excitatory DREADDs were bilaterally expressed in short axon cells using AAV2-DIO-hM3D(Gq)-mCherry (Addgene, 44361-AAV2) in *DAT-Cre* mice, or in cholinergic cells in HDB of *ChAT-Cre* mice. Inhibitory DREADDs were expressed bilaterally to infect *ChAT* fibers projecting to the OB or all *ChAT* neurons in the HDB using AAVrg-DIO-hM4D(Gi)-mCherry (Addgene, 44362-AAVrg) and AAV2-hSyn-DIO-hM4DGi-mCherry (Addgene, 44362-AAV2) in *ChAT-Cre* mice respectively. Inhibitory DREADDs were expressed bilaterally to infect SACs using AAV2-hSyn-DIO-hM4DGi-mCherry (Addgene, 44362-AAV2) in *DAT-Cre* mice. Control experiments involved *DAT-Cre* mice injected with AAV1-CAG-LSL-tdTomato (Addgene, 100048-AAV1) and *ChAT-Cre* mice injected with AAVrg-CAG-FLEX-tdTomato (Addgene, 51503-AAVrg) in the olfactory bulb, as well as AAV1-CAG-LSL-tdTomato in the HDB. Clozapine N-oxide (CNO, Abcam, ab141704) was prepared in 1× PBS and administered intraperitoneally at a dose of 2.5 mg/kg, 20 min prior to all behavioral and imaging experiments.

### TRAP2 expression and signal quantification

4-hydroxy tamoxifen (4-OHT, MilliporeSigma) was dissolved in DMSO and diluted with PBS to a concentration of 2 mg/ml. Animals were injected with 4-OHT at a dose of 30 mg/kg prior to training session or odor exposure. Brain samples were prepared as described above. Slidescanner images were acquired at 10× magnification and binned 2 × 2. Images were aligned to Allen brain atlas using Aligning Big Brains and Atlases (ABBA) and Fos positive signals were quantified in QuPath using custom scripts [82]. Experimenters were blinded during TRAP2 brain alignment and cell segmentation in QuPath.

### Image processing and analysis

Custom scripts developed in ImageJ and MATLAB (MathWorks) were utilized for processing the imaging data, following methodologies previously outlined [31,83]. Regions of interest (ROIs) were semi-automatically delineated using thresholded response images. For longitudinal analyses, images were *z*-projected to generate mean intensity projections, which were then aligned using procedures for angular rotation and translation in ImageJ. Non-overlapping ROIs were collectively analyzed, and the average fluorescence response within each ROI was quantified via batch processing in ImageJ. For imaging of glomerular calcium activity, ROIs were defined as individual glomeruli identifiable by structural boundaries visible under a 4× objective on a widefield microscope. Glomeruli were selected based on their circular morphology and the uniformity of GCaMP fluorescence intensity across the structure. For SAC and MTC dendritic fields, individual ROIs were identifiable by structural boundaries that typically encompassed single glomerulus. Image stacks were manually tracked for intensity variations across odor delivery period to segment nearby ROIs. Manual adjustments were made to exclude overlapping or incomplete glomeruli structures. For SAC soma imaging, ROIs were defined as individual neurons visible under a 20× objective on a two-photon microscope. The consistency of ROI selection across samples was ensured by standardized threshold settings maintained throughout the experiment.

### Response dynamics

Custom MATLAB routines were employed to establish a baseline from pre-stimulus intervals and compute the dF/F. For peak response estimation, a moving average (300 ms) was performed for each trace. Peak responses within the stimulus period were identified as the maximum value in the smoothed trace. These data were then used to compute the mean and standard error of the mean across subjects, facilitating the plotting of average responses. ROI responsiveness during behavioral sessions was determined by setting a detection threshold at three times the standard deviation (SD) of the baseline noise. The selectivity index for each ROI was calculated as the difference between peak responses between trial types divided by the SD of all responses. Selectivity value >1 or <−1 was labelled high and (−1, 1) was labelled low. For temporal dynamics, the SD of each ROI was computed over a 2-s pre-stimulus period to establish a baseline variability. The onset latency was defined as the first frame post-stimulus where the subsequent four frames consecutively exceeded three times the SD. To assess significant changes in response for each glomerulus, we pooled all trials and randomized the trial identities 1,000 times to create a null distribution for each type of modulation. We then performed a *t* test on each glomerulus, applying the Benjamini-Hochberg False Discovery Rate correction for multiple comparisons. Glomeruli with a *p*-value < 0.05 were considered significantly modulated.

### Spatial distribution of ROIs

To quantify the spatial distribution of odor-evoked activations within the OB, we calculated the distance and angular position of each activated region relative to the anatomical centroid of the OB. An oval-shaped region of interest encompassing the OB surface was manually drawn in ImageJ for each animal, and the centroid of the OB was computed with the centroid function in MATLAB. Activation coordinates, corresponding to odor-evoked regions, were then re-centered relative to the OB centroid by subtracting the centroid coordinates from each activation point. For each re-centered activation point, the Euclidean distance from the centroid was calculated and normalized to the maximum radius observed for that animal’s OB.

### Quantification and statistical analysis

Significance was defined at *p* < 0.05 for all statistical tests used in this study. *, ** and *** indicate differences of *p* < 0.05, *p* < 0.01, and *p* < 0.001, respectively. OriginPro (OriginLab) and MATLAB (MathWorks) were used to calculate statistical significance. Summary of statistics is provided in S3 Table. Specific statistical tests employed include the one-sample *t* test to assess whe*t*her mean values were significantly different from zero, and the Wilcoxon rank-sum test (two-tailed) for TRAP2 comparisons. When multiple groups were compared, two-way ANOVA was applied with Tukey’s post hoc test. A mixed-design ANOVA was performed with group as the between-subject factor and condition as the within-subject factor (repeated measures). Additionally, two-sample *t*-tests (two-tailed) and the Kruskal-Wallis test followed by Dunn’s post-hoc test were used for comparison amongst 2 and 3 independent groups respectively. Repeated measures ANOVA was employed where multiple measurements were taken from the same experimental group. All data are presented as mean ± standard error of the mean.

## Supporting information

S1 Fig Learning preferentially enhances reward associated input. **(A, B)** Bar plots show lick frequency **(A)** and CR in discrimination **(B)** over multiple sessions (*n* = 6). *P < 0.05, **P < 0.01, one-way repeated measures ANOVA followed by Tukey’s *post hoc* test (CS− lick frequency: *q*(5) = 4.8, *p* = 0.019; CR: *q*(5) = 7.2, *p* = 0.0037). **(C)** Density of cFos expression across the olfactory bulb post methyl butyrate exposure. *P < 0.05, **P < 0.01, one-way repeated measures ANOVA followed by Tukey’s *post hoc* test (dorsal versus medial *q*(15) = 6.76, *p* = 0.0012; dorsal versus lateral *q*(15) = 5.24, *p* = 0.01; dorsal versus medial *q*(15) = 5.85, *p* = 0.0043). **(D)** Glomeruli response map of individual animals, scale bar: 200 µm. **(E)** Fraction of activated glomeruli in odor selective zones pre and post learning. Two-way repeated measures ANOVA followed by Tukey’s *post hoc* test (Training related increase in CS+ selective glomeruli *q*(5) = 0.003, *p* = 0.99; CS− selective glomeruli *q*(5) = 0.22, *p* = 0.87). **(F)** Distribution of glomerular selectivity. **(G)** Cumulative plot of response onset latency for CS+ (blue) and CS− (brown) odors pre- (left) and post-training (right). One-way repeated measures ANOVA followed by Tukey’s *post hoc* test (Day 2: *q*(12) = 5.82, *p* = 0.0014); Data are mean ± SEM. **(H)** Difference in onset latency between pre and post odor association for same glomerulus. Both CS+ (blue) and CS− (brown) odors are detected faster when mice are actively discriminating odors in task. Pie charts show the number of glomeruli with significant difference from permutation test. Glomeruli with non-significant changes are not included in the pie chart **(I)** Average onset of glomerular response pre and post training segregated by glomerular selectivity and odor valence. *P < 0.05, two-way repeated measures ANOVA followed by Tukey’s *post hoc* test (*q*(5) = 3.74, *p* = 0.04). Data for this figure are provided in S1 Data.
(TIF)

S2 Fig Learning dependent changes across layers of main olfactory bulb. **(A)** GAD1 expression in OB clusters. Magnified region shows GL subcluster and expression of key interneuron markers. **(B)** Distribution of OB interneurons across OB layers. **(C–E)** Distribution of OB cells expressing key synaptic markers. **(F)** Individual density plots show spatial density of TH immunofluorescence over the dorsal OB across different training paradigms (average data in Fig 2E). **(G)** Example image of M72-GFP glomerulus (white) innervated by TH positive cells (yellow). Scale bar 100 µm. **(H–J)** Density **(H)**, counts of TH+ somas **(I)** and volume of M72-GFP glomeruli **(J)** across different training paradigms. *P < 0.05, two-way ANOVA followed by Tukey’s *post hoc* test (Discrimination learning, density: *q*(107) = 4.53, *p* = 0.036; counts: *q*(107) = 4.73, *p* = 0.026). Bold font indicates acetophenone pairing. Data for this figure are provided in S1 Data.
(TIF)

S3 Fig Dynamics of GL fibers with learning. **(A)** Widefield imaging of SAC dendritic fibers using GCaMP7F. Bottom: example virus transduction, scale bar: 50 µm. **(B)** Average calcium traces odors recorded across the dorsal surface in passively exposed animals. (*n* = 12). **(C)** Bar graph shows average peak responses. N.S, one-way repeated measures ANOVA followed by Tukey’s *post hoc* test. **(D)** Onset latency for individual ROIs on the dendritic field (left) and somas (right) for the SACs across odor valence. ***P < 0.001. Two-way repeated measures ANOVA followed by Tukey’s *post hoc* test (soma: *q*(18) = 16.21, *p* < 0.0001). **(E–G)** Same as **(A–C)** but for MTC dendrites (*n* = 14 animals for passive and trained conditions each). **P < 0.01, ***P < 0.001, mixed-design ANOVA followed by Tukey’s *post hoc* test (Trained: *q*(26) = 4.05, *p* = 0.008). Data for this figure are provided in S1 Data.
(TIF)

S4 Fig Bidirectional SAC perturbation using chemogenetics. **(A)** Schematic for Gq and GCaMP7f expression in SACs for simultaneous imaging and chemogenetics. Bottom: Example colocalization of Gq (red) with GCaMP7 (green), scale bar: 200 µm. **(B)** Widefield imaging of SAC fibers post Saline and CNO injections in Gq injected animal, scale bar: 200 µm. **(C)** Peak odor-driven responses in SAC fibers with Saline and CNO injections. One-way repeated measures ANOVA followed by Tukey’s *post hoc* test (*q*(323) = 7.58, *p* < 0.0001). **(D, E)** Density of viral spread across dorsal OB for mice used for Fig 4H–4J. **(F)** Fraction of activated glomeruli across dorsal OB for CS+ (left) and CS− (right) odors in mCherry and Gq injected animals post CNO injections. **(G)** Schematics for Gi and GCaMP7f expression in SACs for simultaneous imaging and chemogenetics. **(H)** Widefield imaging of SAC fibers post Saline and CNO injections in Gi injected animal, scale bar: 200 µm. **(I)** Peak odor-driven responses in SAC fibers with Saline and CNO injections. One-way repeated measures ANOVA followed by Tukey’s *post hoc* test (*q*(235) = 8.92, *p* < 0.0001). **(J)** Strategy for inhibition of SACs using Gi expression. **(K)** Accuracy in odor association in mCh and Gi injected animals post Saline (Pair-1) and CNO (Pair-2) injections. **P < 0.01, ***P < 0.001, mixed-design ANOVA followed by Tukey’s *post hoc* test (Gi: *q*(14) = 4.83, *p* = 0.0041). Data for this figure are provided in S1 Data.
(TIF)

S5 Fig Brain-wide c-fos expression and perturbation of HDB◊OB inputs using chemogenetics. **(A)** Odor learning associated brain areas identified using TRAP2;Ai14 line. **(B)** Proportion of cholinergic cells in HDB labelled with retrograde Gi virus in OB of mice used for Fig 6B–6D. **(C)** Fraction of activated glomeruli across dorsal OB for CS+ (left) and CS− (right) odors in mCherry and Gi injected animals post CNO injections. **(D)** Accuracy in discrimination of same odor pair post-Saline (Session-1) and CNO (Session-2) injections in mCh (left, *n* = 7) and Gi (right, *n* = 8) animals. **P < 0.01, mixed-design ANOVA followed by Tukey’s post hoc test (Session-2 mCh versus Gi: *q*(13) = 6.12, *p* = 0.0039). **(E)** Spatial density of TH immunofluorescence over the dorsal OB after odor training in mCh (left) and Gi (right) animals. **P < 0.01, two sample *t* test (two-tailed; *t*(6) = 4.05, *p* = 0.0067). Data for this figure are provided in S1 Data.
(TIF)

S1 Table Odorants used in this study. (XLSX)

S2 Table List of genes probed by Molecular Cartography. (XLSX)

S3 Table Summary of statistics. (XLSX)

S1 Data All tabulated data. (XLSX)

## Abbreviations

**:**
AAV: adeno-associated virus
OB: olfactory bulb
OSN: olfactory sensory neurons
ROI: region of interest
SAC: short axons cell
HDB: horizontal limb of the diagonal band
