# Supplementary figures and images for: Dopaminergic short axon cells integrate sensory and top–down inputs to enhance discriminative learning in the mouse olfactory bulb
Source: PLoS Biol. 2025 Sep 16;23(9):e3003375. doi: 10.1371/journal.pbio.3003375 (PMC12440180; doi:10.1371/journal.pbio.3003375)

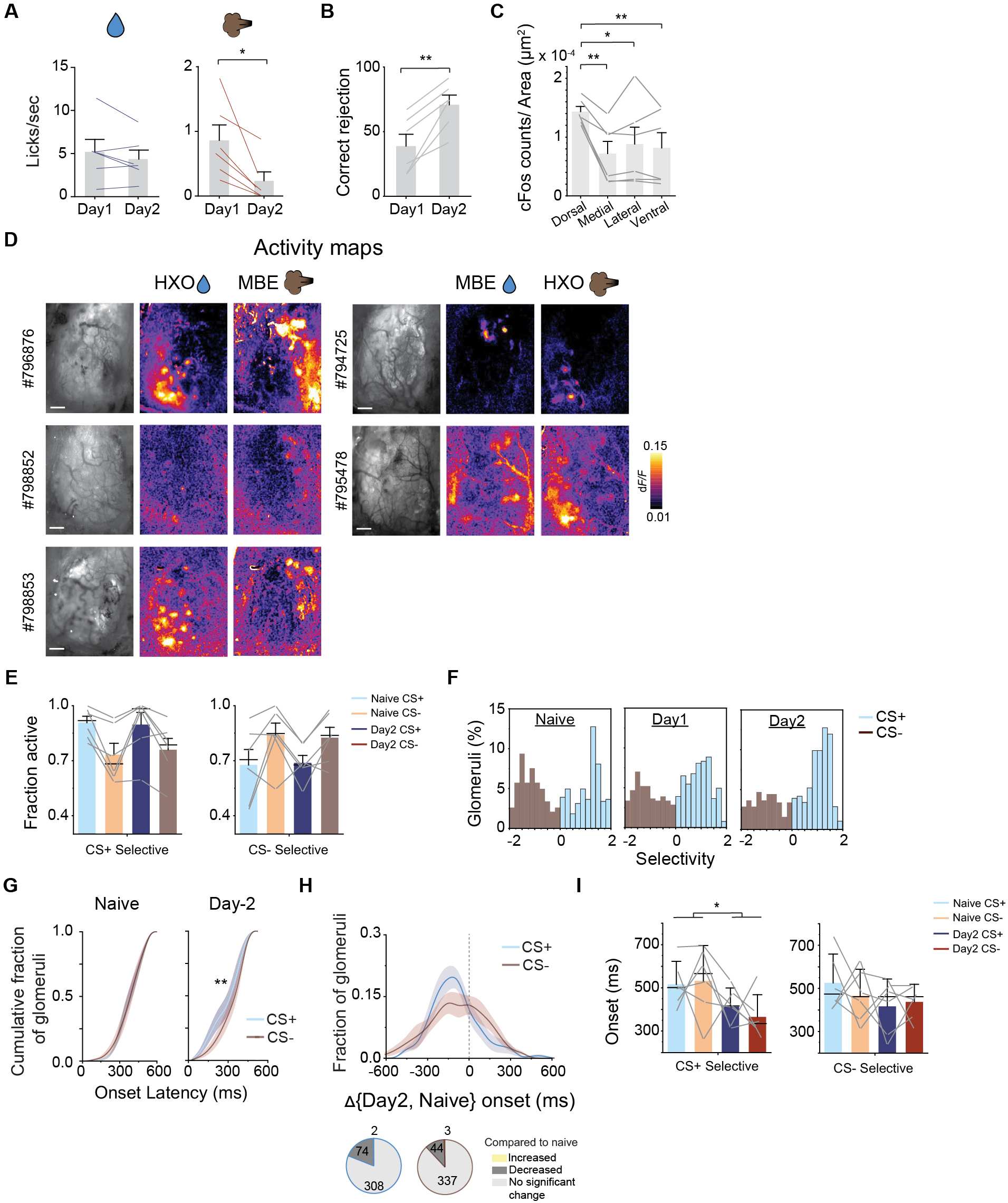

Supplement: S1 Fig — (A, B) Bar plots show lick frequency (A) and CR in discrimination (B) over multiple sessions (n = 6). *P < 0.05, **P < 0.01, one-way repeated measures ANOVA followed by Tukey’s post hoc test (CS− lick frequency: q(5) = 4.8, p = 0.019; CR: q(5) = 7.2, p = 0.0037). (C) Density of cFos expression across the olfactory bulb post methyl butyrate exposure. *P < 0.05, **P < 0.01, one-way repeated measures ANOVA followed by Tukey’s post hoc test (dorsal versus medial q(15) = 6.76, p = 0.0012; dorsal versus lateral q(15) = 5.24, p = 0.01; dorsal versus medial q(15) = 5.85, p = 0.0043). (D) Glomeruli response map of individual animals, scale bar: 200 µm. (E) Fraction of activated glomeruli in odor selective zones pre and post learning. Two-way repeated measures ANOVA followed by Tukey’s post hoc test (Training related increase in CS+ selective glomeruli q(5) = 0.003, p = 0.99; CS− selective glomeruli q(5) = 0.22, p = 0.87). (F) Distribution of glomerular selectivity. (G) Cumulative plot of response onset latency for CS+ (blue) and CS− (brown) odors pre- (left) and post-training (right). One-way repeated measures ANOVA followed by Tukey’s post hoc test (Day 2: q(12) = 5.82, p = 0.0014); Data are mean ± SEM. (H) Difference in onset latency between pre and post odor association for same glomerulus. Both CS+ (blue) and CS− (brown) odors are detected faster when mice are actively discriminating odors in task. Pie charts show the number of glomeruli with significant difference from permutation test. Glomeruli with non-significant changes are not included in the pie chart (I) Average onset of glomerular response pre and post training segregated by glomerular selectivity and odor valence. *P < 0.05, two-way repeated measures ANOVA followed by Tukey’s post hoc test (q(5) = 3.74, p = 0.04). Data for this figure are provided in S1 Data. (TIF) [file pbio.3003375.s001.tif]

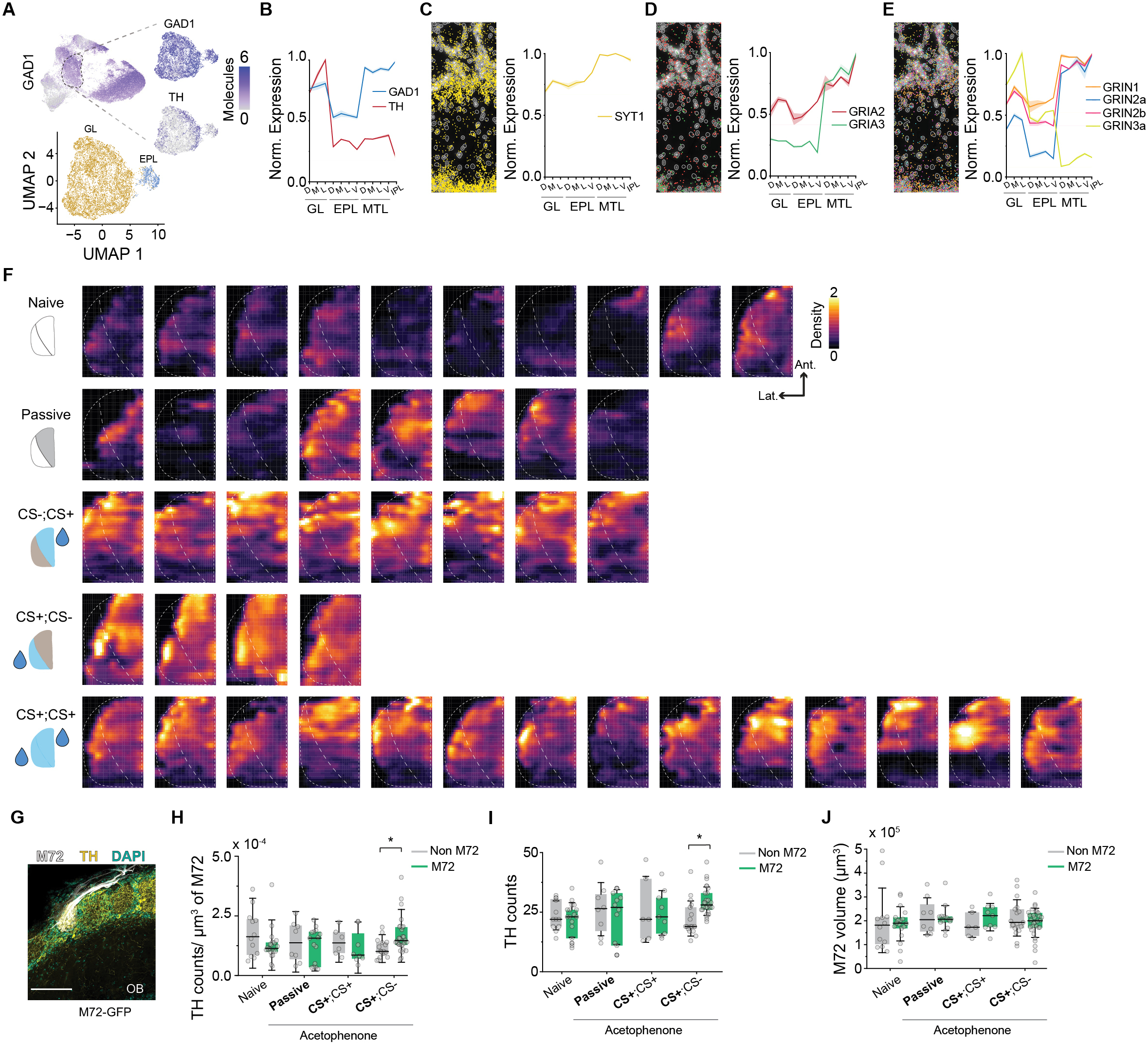

Supplement: S2 Fig — (A) GAD1 expression in OB clusters. Magnified region shows GL subcluster and expression of key interneuron markers. (B) Distribution of OB interneurons across OB layers. (C–E) Distribution of OB cells expressing key synaptic markers. (F) Individual density plots show spatial density of TH immunofluorescence over the dorsal OB across different training paradigms (average data in Fig 2E). (G) Example image of M72-GFP glomerulus (white) innervated by TH positive cells (yellow). Scale bar 100 µm. (H–J) Density (H), counts of TH+ somas (I) and volume of M72-GFP glomeruli (J) across different training paradigms. *P < 0.05, two-way ANOVA followed by Tukey’s post hoc test (Discrimination learning, density: q(107) = 4.53, p = 0.036; counts: q(107) = 4.73, p = 0.026). Bold font indicates acetophenone pairing. Data for this figure are provided in S1 Data. (TIF) [file pbio.3003375.s002.tif]

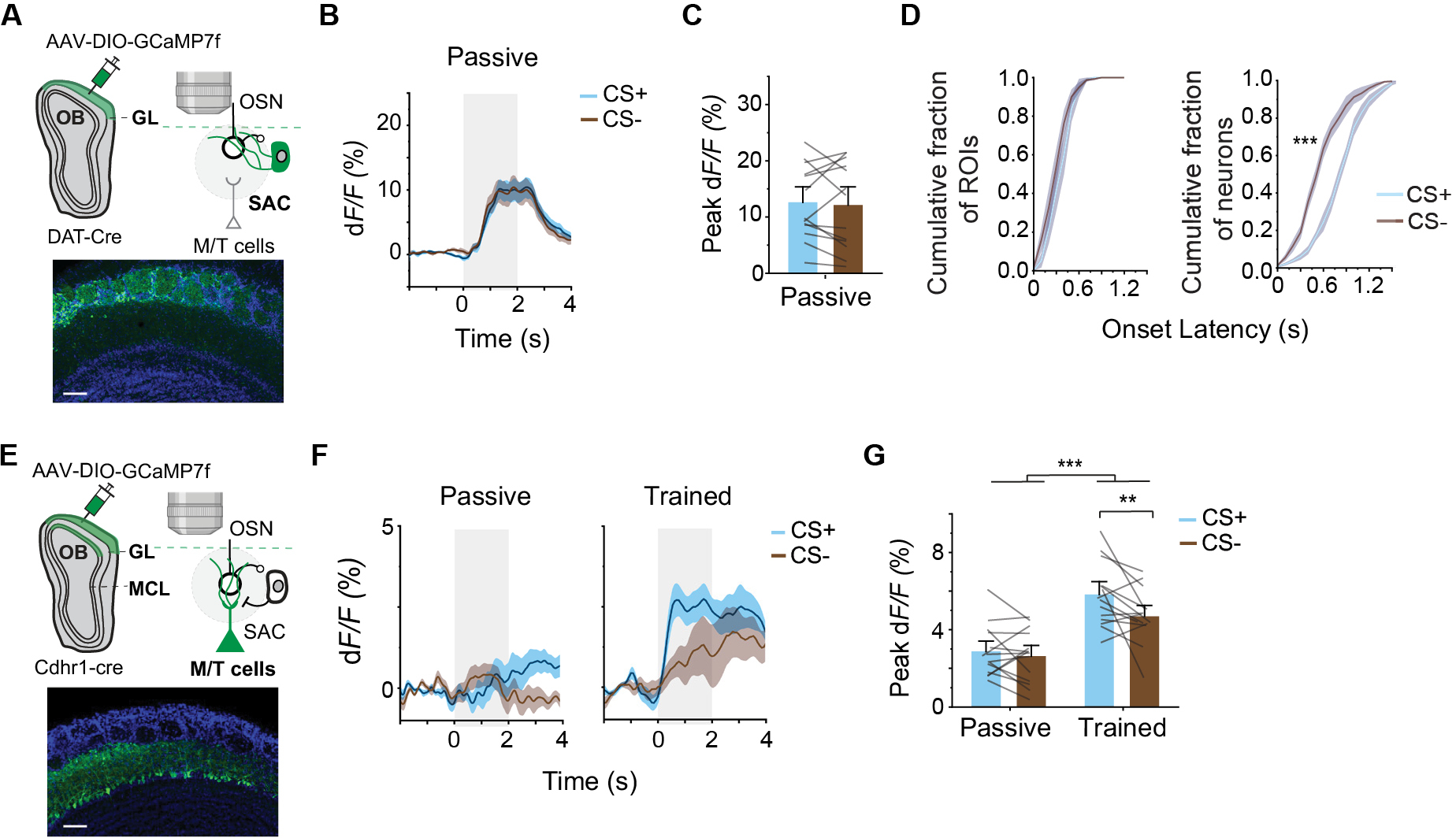

Supplement: S3 Fig — (A) Widefield imaging of SAC dendritic fibers using GCaMP7F. Bottom: example virus transduction, scale bar: 50 µm. (B) Average calcium traces odors recorded across the dorsal surface in passively exposed animals. (n = 12). (C) Bar graph shows average peak responses. N.S, one-way repeated measures ANOVA followed by Tukey’s post hoc test. (D) Onset latency for individual ROIs on the dendritic field (left) and somas (right) for the SACs across odor valence. ***P < 0.001. Two-way repeated measures ANOVA followed by Tukey’s post hoc test (soma: q(18) = 16.21, p < 0.0001). (E–G) Same as (A–C) but for MTC dendrites (n = 14 animals for passive and trained conditions each). **P < 0.01, ***P < 0.001, mixed-design ANOVA followed by Tukey’s post hoc test (Trained: q(26) = 4.05, p = 0.008). Data for this figure are provided in S1 Data. (TIF) [file pbio.3003375.s003.tif]

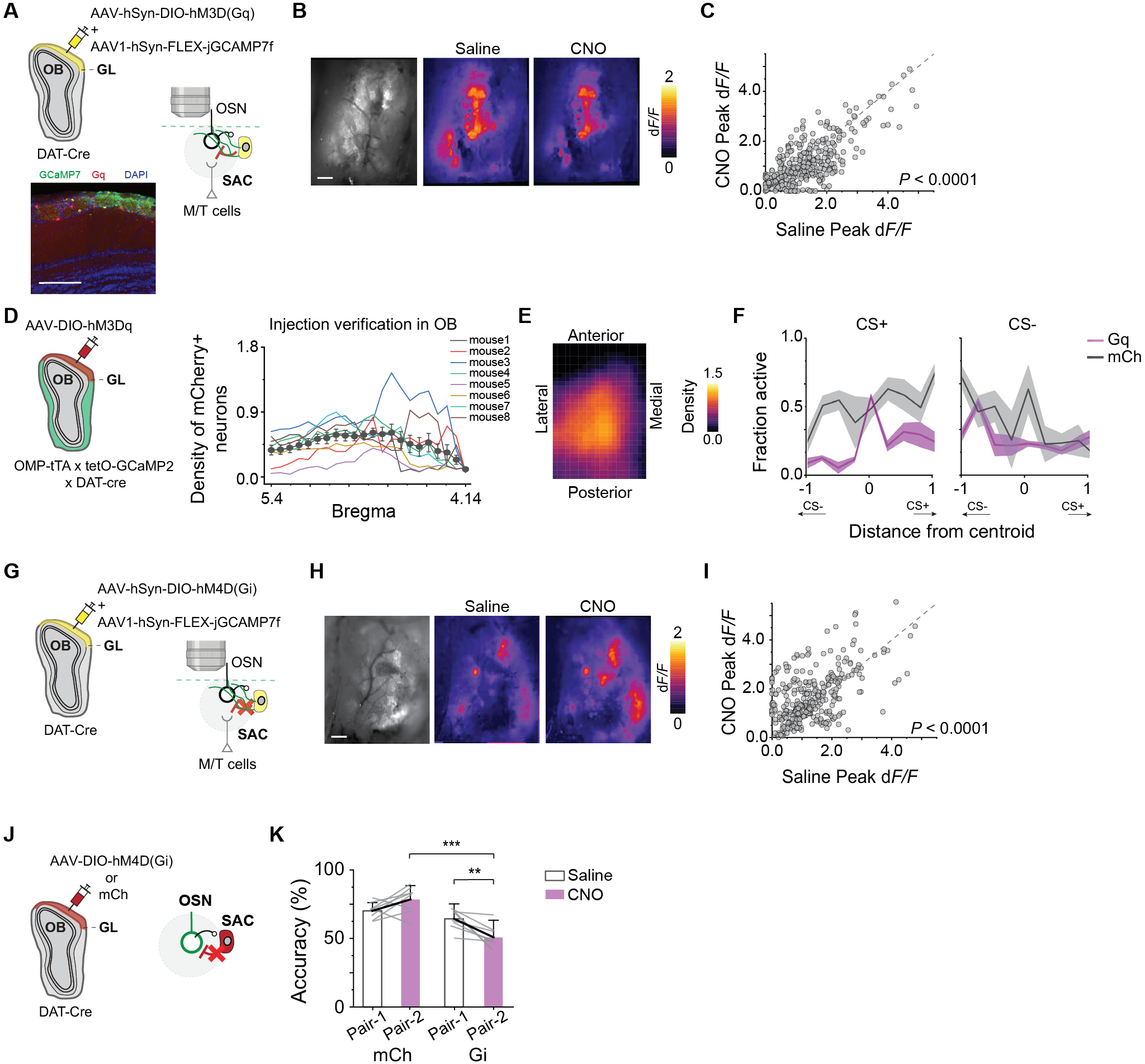

Supplement: S4 Fig — (A) Schematic for Gq and GCaMP7f expression in SACs for simultaneous imaging and chemogenetics. Bottom: Example colocalization of Gq (red) with GCaMP7 (green), scale bar: 200 µm. (B) Widefield imaging of SAC fibers post Saline and CNO injections in Gq injected animal, scale bar: 200 µm. (C) Peak odor-driven responses in SAC fibers with Saline and CNO injections. One-way repeated measures ANOVA followed by Tukey’s post hoc test (q(323) = 7.58, p < 0.0001). (D, E) Density of viral spread across dorsal OB for mice used for Fig 4H–4J. (F) Fraction of activated glomeruli across dorsal OB for CS+ (left) and CS− (right) odors in mCherry and Gq injected animals post CNO injections. (G) Schematics for Gi and GCaMP7f expression in SACs for simultaneous imaging and chemogenetics. (H) Widefield imaging of SAC fibers post Saline and CNO injections in Gi injected animal, scale bar: 200 µm. (I) Peak odor-driven responses in SAC fibers with Saline and CNO injections. One-way repeated measures ANOVA followed by Tukey’s post hoc test (q(235) = 8.92, p < 0.0001). (J) Strategy for inhibition of SACs using Gi expression. (K) Accuracy in odor association in mCh and Gi injected animals post Saline (Pair-1) and CNO (Pair-2) injections. **P < 0.01, ***P < 0.001, mixed-design ANOVA followed by Tukey’s post hoc test (Gi: q(14) = 4.83, p = 0.0041). Data for this figure are provided in S1 Data. (TIF) [file pbio.3003375.s004.tif]

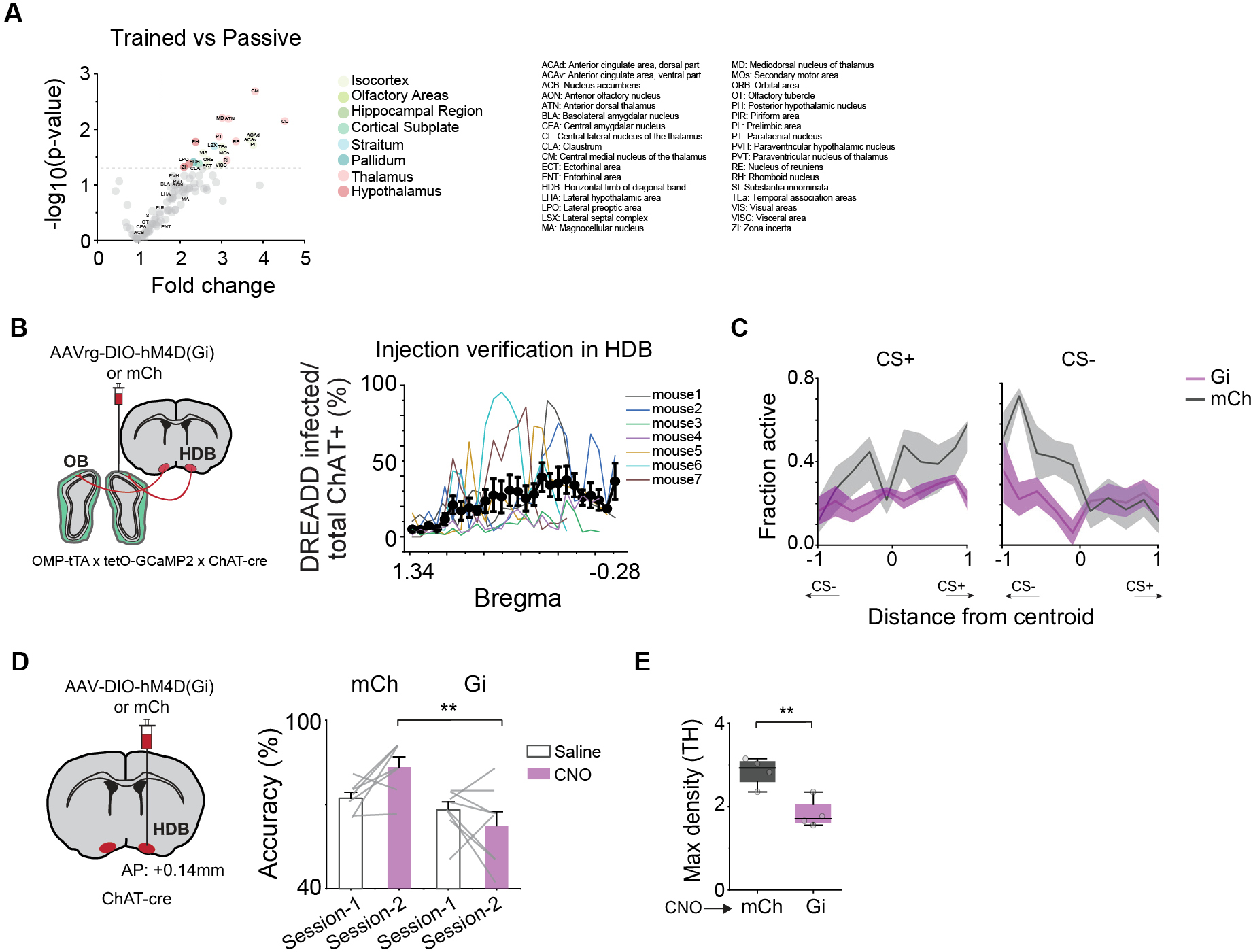

Supplement: S5 Fig — (A) Odor learning associated brain areas identified using TRAP2;Ai14 line. (B) Proportion of cholinergic cells in HDB labelled with retrograde Gi virus in OB of mice used for Fig 6B–6D. (C) Fraction of activated glomeruli across dorsal OB for CS+ (left) and CS− (right) odors in mCherry and Gi injected animals post CNO injections. (D) Accuracy in discrimination of same odor pair post-Saline (Session-1) and CNO (Session-2) injections in mCh (left, n = 7) and Gi (right, n = 8) animals. **P < 0.01, mixed-design ANOVA followed by Tukey’s post hoc test (Session-2 mCh versus Gi: q(13) = 6.12, p = 0.0039). (E) Spatial density of TH immunofluorescence over the dorsal OB after odor training in mCh (left) and Gi (right) animals. **P < 0.01, two sample t test (two-tailed; t(6) = 4.05, p = 0.0067). Data for this figure are provided in S1 Data. (TIF) [file pbio.3003375.s005.tif]
